# *Turicibacter sanguinis* is a candidate gut microbial pathobiont that promotes metabolic dysfunction-associated steatohepatitis

**DOI:** 10.1128/msystems.00292-26

**Published:** 2026-05-18

**Authors:** Jing Guo, Zhong-wen Xiang, Fang-fang Hu, Song-xia Zhang, Wen-jing Han, Xin Ding, Xin Wang, Meng-ling Ye, Jun-hong Chen, Tai Rao, Lie-lin Wu, Guang-hui Lian, Wei Zhang, Yun Huang, Yao Chen

**Affiliations:** 1 Department of Clinical Pharmacology, National Clinical Research Center for Geriatric Disease, Xiangya Hospital, Central South University 159374, Changsha, Hunan, China; 2 Department of Organ Transplantation, Xiangya Hospital, Central South University 159374, Changsha, Hunan, China; 3 Department of Gastroenterology, Xiangya Hospital, Central South University 159374, Changsha, Hunan, China; 4 Department of Hepatobiliary Surgery, Xiangya Hospital, Central South University 159374, Changsha, Hunan, China; Johns Hopkins University Bloomberg School of Public Health, Baltimore, Maryland, USA

**Keywords:** MASH, *Turicibacter sanguinis*, bile acid, FXR

## Abstract

Emerging evidence points to the gut microbiota’s involvement in metabolic dysfunction-associated steatohepatitis (MASH), yet the specific causative microbes remain largely unidentified. This study aimed to identify and functionally characterize candidate microbial pathobionts to MASH progression. Differentially abundant microbes were identified by 16S rRNA sequencing in a choline-deficient, L-amino acid-defined, high-fat diet MASH model, validated in other animal MASH models and in public clinical metagenomic data sets, then screened for consistently altered gut taxa. A candidate underwent functional validation via directed oral administration in mice. Mechanisms were explored through bile acid profiling by UHPLC-MS/MS and FXR signaling analysis by qPCR and immunohistochemistry. Additionally, fecal samples from MASH patients before and after treatment were analyzed to correlate microbial abundance with treatment response. *Turicibacter sanguinis* was consistently enriched in all MASH models and public data sets, with abundance correlating positively with liver injury markers. Its increased abundance exacerbated steatosis, inflammation, and fibrosis in healthy and diseased mice. Mechanistically, *Turicibacter sanguinis* altered bile acid composition, thereby increasing conjugated and decreasing unconjugated species, and inhibited hepatic FXR signaling, accompanied by suppressed SHP and elevated CYP7A1 and SREBP1c expression, which is consistent with enhanced bile acid synthesis and lipid accumulation. Futhermore, after pharmacotherapy, reduced *Turicibater sanguinis* levels correlated positively with alanine aminotransferase (ALT) and aspartate aminotransferase (AST) improvements. In conclusion, *Turicibacter sanguinis* is a clinically relevant microbial pathogen that exacerbated MASH by inducing bile acid dysregulation and suppressing FXR signaling, highlighting its potential as a candidate biomarker for disease monitoring and motivating future evaluation of targeted microbiome interventions.

**IMPORTANCE:**

Metabolic dysfunction-associated steatohepatitis (MASH) is a growing global health problem with limited treatment options. Although the gut microbiome has been implicated in MASH, the specific bacterial strains that directly drive disease progression remain largely unknown. This study identified *Turicibacter sanguinis* as a candidate gut microbial pathobiont that promotes MASH, demonstrating its significant enrichment in both animal models and patient samples. By disrupting hepatic metabolic signaling, this bacterium promotes bile acid synthesis and exacerbates liver fat accumulation, inflammation, and fibrosis. Following effective treatment, its abundance decreased significantly in patients. These findings indicate that *Turicibacter sanguinis* holds promise as a potential target for developing novel microbiome-based diagnostic and therapeutic approaches for MASH.

## INTRODUCTION

Metabolic dysfunction-associated steatohepatitis (MASH) represents a progressive form of metabolic dysfunction-associated steatotic liver disease (MASLD), marked by the onset of liver inflammation and subsequent damage (1, 2). Epidemiological surveys indicate that roughly 38% of the global population has hepatic steatosis (3), and approximately one-fifth of these individuals progress to MASH (4, 5). Consequently, MASH has become a leading global health challenge. Although the recent approval of the thyroid-hormone-receptor-β agonist Resmetirom marks a therapeutic milestone (6), effective options remain scarce, largely because of a paucity of druggable targets. While classic theories, such as the “two-hit hypothesis” (7), have laid an important foundation for understanding the pathogenesis of MASH, they still fall short of fully revealing its complexity. Therefore, there is an urgent need for a more comprehensive perspective to deepen the understanding of MASH upon existing knowledge and accelerate the development of novel treatment strategies.

Emerging evidence underscores the gut-liver axis as a central driver of MASH pathogenesis (8). Boasting a genetic repertoire far surpassing that of humans (9), the gut microbiome directly shapes hepatic metabolism and immunity via its metabolites and antigens (10). Clinical metagenomic studies reveal that MASH patients exhibit depletion of Firmicutes alongside enrichment of endotoxin-producing Pseudomonadota and bile-acid-regulating Bacteroidetes (11–13). This dysbiosis compromises the intestinal barrier, allowing microbial products like lipopolysaccharide, peptidoglycan, and bacterial DNA to leak into the portal circulation. This process, in turn, amplifies key pathological drivers such as oxidative stress, *de novo* lipogenesis, and stellate-cell activation (14, 15). Moreover, microbiota-derived bile acids and others can modulate host signaling cascades to promote hepatic lipid accumulation and inflammation (16, 17). In recent years, microbial interventions have shown therapeutic promise: supplementation with *Bacteroides thetaiotaomicron* confers protection against hepatic steatosis (18), and *Bacteroides xylanisolvens* mitigates smoking-aggravated MASH by breaking down nicotine (19). Conversely, the pathobiont *Blautia producta* has been identified as a driver of MASH fibrosis (20). These findings reveal the therapeutic promise of precise gut microbiota modulation for MASH. Despite these advances, most evidence remains correlative, and the functional impacts and causal mechanisms of individual bacterial strains, particularly pathobionts, are still poorly understood. Thus, there is a pressing need to functionally validate the roles of key bacteria in MASH pathogenesis.

This study found that a selective enrichment of *Turicibacter* in a choline-deficient, L-amino acid-defined, high-fat diet (CDAHFD) induced MASH model, and subsequently confirmed in other MASH models, as well as in publicly available clinical metagenomic data sets. Among the differentially abundant taxa, *Turicibacter* was the only genus consistently altered across all systems. As a Gram-positive bacterium within the phylum *Firmicutes*, the genus *Turicibacter* is increasingly implicated in metabolic disorders, with studies reporting its association despite its relatively minor abundance (~0.5%) in the human gut microbiota (21, 22). As a dominant species within this genus, *Turicibacter sanguinis* (*T. sanguinis*) may represent a candidate pathobiont and therapeutic target for MASH. However, whether it plays a causal role in MASH pathogenesis has yet to be elucidated. Based on these findings, this study intended to employ *T. sanguinis* administration experiments to validate its pathogenic role in MASH at the *in vivo* level. Further investigations included bile acid profiling and analysis of the FXR signaling pathway to elucidate the underlying mechanisms. Concurrently, changes in the abundance of *T. sanguinis* were assessed in clinical samples following interventional therapy to explore its biomarker and therapeutic target potential. This study sought to elucidate the role of *T. sanguinis* in MASH and to provide a rational target for precision microbiome-based therapies.

## MATERIALS AND METHODS

### Screening for MASH-associated gut microbiota in CDAHFD models and validating in other models

To uncover core MASH-associated gut microbes independent of specific models, initial screening was performed using the CDAHFD-induced MASH model, followed by validation in other models to screen microbial taxa consistently enriched across diverse metabolic contexts.

#### Animal MASH model

Male C57BL/6J mice (Hunan SJA Laboratory Animal Co., Ltd., China) were maintained under standard conditions: a 12-h photoperiod, temperature of 22°C ± 2°C, and provided with autoclaved water and irradiated chow *ad libitum*. All mice were euthanized by cervical dislocation under deep anesthesia induced by exposure to 4% isoflurane in an inhalation chamber.

Three distinct MASH models were established, including the CDAHFD, MCD, and HFD-induced models, which are described below. The MCD and HFD experiments were conducted in the same vivarium, whereas the CDAHFD experiment was performed in a separate animal facility under independent housing conditions. CDAHFD model: an 8-week feeding period with CDAHFD (0.1% methionine, 0% choline, 60% fat), a normal diet as the control. MCD model: a 5-week feeding period with MCD, with a methionine- and choline-supplemented diet as the control. HFD model: an 18-week feeding period with HFD (42% fat, 0.2% cholesterol), with a low-fat diet (LFD, 10% fat) as the control. All diets were purchased from Trophic (Nantong, China). Tissue samples were collected at the experimental endpoint.

#### 16S rRNA sequencing

Upon successful model establishment, mice feces were subjected to 16S rRNA gene sequencing for microbiota analysis. DNA was extracted (TIANamp Stool DNA Kit), the V4 region amplified (515F/806R primers), and amplicons were gel-purified (GeneJET Kit). Libraries were prepared (TruSeq DNA PCR-Free Kit) and sequenced on an Illumina NovaSeq 6000. Raw sequencing data were processed using QIIME2 (version 2022.2). After quality filtering and chimera removal, sequences were clustered into OTUs at 97% similarity, and taxonomic assignment was performed using the SILVA reference database (version 138.1). For the CDAHFD model, the average number of reads per sample was approximately 9.1 × 10⁴, ranging from 83,894 to 99,015 reads. For the MCD model, the average number of reads per sample was approximately 7.8 × 10⁴, ranging from 68,860 to 80,225 reads. For the HFD model, the average sequencing depth was approximately 5.4 × 10⁴ reads per sample, with a range of 42,676 to 69,698 reads. To minimize bias introduced by uneven sequencing depth, feature tables were rarefied to the minimum sequencing depth within each cohort prior to alpha and beta diversity analyses. To identify differentially abundant taxa between each model group and its corresponding control group, linear discriminant analysis effect size (LEfSe) was employed with the default parameters (LDA score threshold > 3.0). Additionally, the Mann-Whitney test was used to compare the relative abundances of taxa. Consistently altered gut bacteria across models were defined as those taxa that were significantly altered in all three models (CDAHFD, MCD, and HFD) and exhibited the same direction of change.

### Validation of gut microbial candidates in public clinical metagenomic database*s*

To explore the clinical relevance of gut microbial candidates found in MASH animal models, public clinical metagenomic data sets were used to validate if these microbes are also abundant in human patients.

#### Bioinformatics analysis

Raw metagenomic sequencing data were obtained from BioProject PRJEB55534. Samples annotated in the original data set as MASH or healthy were included for analysis. No additional matching procedures were performed. After excluding samples that could not be successfully downloaded or processed, a total of 125 samples were retained, including 109 MASH cases and 16 healthy controls (Table S1). The data were processed using KneadData (23) and Bowtie2 (version 2.5.3) (24) for quality control, involving quality filtering and depletion of host-derived sequences. Taxonomic classification and relative abundance estimation of the quality-controlled metagenomic data were performed with Kraken2 (version 2.1.3) (25) and Bracken (version 2.9) (26), generating species composition profiles and abundance metrics at multiple taxonomic levels for each sample. Differential abundance analysis was then performed on the microbial candidates identified in animal models to compare their levels between MASH patients and healthy controls.

### Causal validation of *T. sanguinis* in MASH progression

To further establish causality beyond mere association, this study employed two complementary approaches: depleting the gut microbiota via antibiotics to create a pseudo-sterile mouse model, and performing directed administration with *T. sanguinis*. These strategies were designed to validate the necessity of the gut microbiota in MASH progression and the sufficiency of *T. sanguinis* in driving the disease, respectively.

#### Antibiotic cocktail treatment in mice

For creating a pseudo-sterile mouse model, an antibiotic cocktail (ABX) consisting of vancomycin (125 mg/L), neomycin sulfate, ampicillin, and metronidazole (all 250 mg/L) was administered via drinking water daily to deplete gut microbiota. A total of eighteen C57BL/6J mice were randomly allocated to three dietary regimens: the control group, fed a normal diet; the MASH group, fed CDAHFD; the MASH+ABX group, fed CDAHFD and supplied with ABX-containing drinking water daily. Treatment lasted for 8 weeks.

To confirm gut microbiota depletion, fecal samples were collected from mice, and total bacterial DNA was extracted using the TIANamp Stool DNA Kit, followed by 16S rRNA gene sequencing. The sample collection and data processing procedures for 16S rRNA gene sequencing were the same as described above. The average number of reads per sample was approximately 9.1 × 10⁴, ranging from 83,341 to 99,211.

#### *T. sanguinis* treatment in mice

*T. sanguinis* (DSM14220, DSMZ, Germany) was revived from −80°C glycerol stocks and cultured to the stationary phase in pre-reduced mGAM medium at 37°C under anaerobic conditions. The culture was then centrifuged at ~4,000 rpm for 15 min to pellet the cells, which were washed 1–2 times with sterile, pre-reduced PBS and resuspended to prepare the gavage suspension. For each batch, the suspension was standardized across preparations by measuring OD_600_ (turbidimetric method) and adjusted to a final gavage volume of 200 µL per mouse (the inoculum was estimated based on OD_600_ to be ~1 × 10^9^ cells per dose).

For *T. sanguinis* treatment, mice received *T. sanguinis* by oral gavage once daily for 8 weeks. The experimental conditions included, control group: fed a normal diet; *T. sanguinis* group: fed a normal diet and administered *T. sanguinis*; MASH group: fed CDAHFD; MASH + *T. sanguinis* group: fed CDAHFD and administered *T. sanguinis*. Mice in the *T. sanguinis* and MASH + *T. sanguinis* groups were gavaged daily with the bacterial suspension as described above, whereas the remaining groups received an equal volume of PBS as a vehicle control.

Fresh fecal samples were collected 72 h after the final *T. sanguinis* gavage, with samples from every two mice pooled into one tube. At sacrifice, small intestinal and cecal contents were also collected and similarly pooled (two mice per tube). Total DNA was extracted from all samples, and the abundance of *T. sanguinis* was quantified by quantitative real-time qPCR.

#### Biochemistry analysis

Following sacrifice, serum and liver tissues were harvested for standard biochemical assays and histopathological evaluation. Serum alanine aminotransferase (ALT), aspartate aminotransferase (AST), and hepatic triglyceride (TG) levels were assayed with commercial kits from Nanjing Jiancheng Bioengineering Institute (Nanjing, China). And serum total bilirubin (TBIL) was assayed with commercial kits from Elabscience Biotechnology Co., Ltd. (Wuhan, China). All assays were conducted strictly in accordance with the manufacturer’s protocols.

#### Histological analysis

Following fixation in 4% paraformaldehyde, the liver tissue samples were subjected to routine dehydration and paraffin embedding. Hematoxylin and eosin (H&E) staining was evaluated for liver steatosis, ballooning, and inflammation. Sirius red staining was used to evaluate collagen. Frozen liver tissues were subjected to Oil Red O staining to evaluate lipids. For histopathological analysis, three of the six mice in each group were randomly selected after completion of the intervention as a predefined representative subset for liver tissue evaluation. Liver tissues were fixed, embedded, sectioned, and stained according to standard procedures. All histological slides were evaluated in a blinded manner.

### Mechanistic investigation of *T. sanguinis* in MASH

Based on previous findings that *T. sanguinis* can remodel bile acids and considering the role of FXR in MASH pathogenesis, this study hypothesized that *T. sanguinis* exacerbates disease by disrupting bile acid and inhibiting hepatic FXR signaling. To preliminarily assess this possibility, this study quantified changes in serum bile acid composition using UHPLC-MS/MS and evaluated mRNA and protein alterations in the hepatic FXR pathway via real-time qPCR and immunohistochemistry.

#### Targeted bile acid analysis

The targeted analysis of bile acid was described previously (27). The representative mass spectrum of bile acid is shown in Fig. S1. The 50 μL mice serum was mixed with 1 mL acetonitrile and 10 μL internal standards (IS, 400 ng/mL) solution containing D4-LCA, D4-CA, D4-GCA, and D4-TCA and then mixed by vortexing before centrifugation at 14,000 × *g* for 15 min. After collecting the supernatant and vacuum-drying, it was then prepared for UHPLC-MS/MS detection by reconstitution in 50 μL of 50% methanol.

Quantitative analysis of bile acids was performed using a UHPLC (Shimadzu Nexera X2)-QQQ MS (AB SCIEX 6500+) system. Separation utilized a Waters ACQUITY UPLC HSS T3 column (2.1 × 100 mm, 1.8 μm; 40°C) with a 0.3 mL/min gradient of 10 mM ammonium acetate in acetonitrile/water. Mass spectrometry was performed in negative polarity using a capillary voltage of 4.5 kV. The parameters of the instrument were set as follows: GAS1: 50 psi; GAS2: 60 psi; TEM: 550°C; CUR: 30 psi. Other parameters are provided in Table S2. Detection under MRM mode provided data processed by AB Sciex Analyst Software (version 1.6.3) for peak area calculation.

#### RNA extraction and real-time qPCR analysis

Total RNA was isolated from tissues using TaKaRa RNAiso Plus, followed by cDNA synthesis with the PrimerScript RT reagent Kit (both from TaKaRa, Japan). Gene expression was quantified by three-step qRT-PCR using SYBR Green PCR Master Mix (Selleck, China). All primer sequences are provided in Table S3.

#### Immunohistochemistry

Deparaffinized and rehydrated liver sections underwent heat-induced antigen retrieval in citrate (10 mM, pH 6.0) or EDTA (1 mM, pH 8.0/9.0) buffer. After blocking endogenous peroxidase with 3% H₂O₂ and non-specific sites with 5% normal serum (1 h), sections were incubated with primary antibodies against FXR, SHP, CYP7A1, and SREBP1c at 4°C overnight. Following PBS washes, signals were developed using HRP-conjugated secondary antibodies with DAB chromogen or fluorescence-conjugated antibodies for direct imaging. Nuclei were counterstained with DAPI, and sections were finally dehydrated, cleared, and mounted. Images were acquired by microscopy, and protein expression was quantified using Image J (NIH, USA).

### Clinical evaluation of the association between *T. sanguinis* and therapeutic effect in MASH

#### Patients

To evaluate the clinical relevance of *T. sanguinis* and its correlation with therapeutic effect, this prospective single-center pilot study enrolled 10 MASH patients at Xiangya Hospital, Central South University. Fecal samples and corresponding clinical data were collected at baseline and after 4 weeks of standardized pharmacotherapy with silibinin and vitamin E. Clinical characteristics of the participants are provided in Table S4.

This study adopted the diagnostic criteria from the 2024 Chinese Society of Hepatology guidelines for MAFLD for patient inclusion: imaging findings indicate the presence of fatty liver; presence of at least one metabolic abnormality, including overweight/obesity, hypertension, hyperglycemia, hypertriglyceridemia, or low high-density lipoprotein cholesterol; moderately elevated serum transaminase or gamma-glutamyl transferase levels persisting for more than 4 weeks. Exclusion criteria include a history of excessive alcohol consumption, severe comorbidities, including malignant tumors, cardiopulmonary diseases, etc., and the receipt of antibiotics or probiotics within 2 weeks pre-enrollment or during the trial.

Fecal samples were collected under standardized conditions, immediately snap-frozen in liquid nitrogen after collection, and stored at −80°C until analysis. Metagenomic sequencing was performed to determine gut microbial composition, and the relative abundance of *T. sanguinis* was quantified based on species-level taxonomic profiling of shotgun metagenomic data. Clinical and biochemical parameters, including liver injury markers (ALT, AST, γGT, TBIL) and serum lipid (TG, TC, HDL-c, LDL-c), were assessed at baseline and treated MASH.

#### Metagenomic sequencing

Total DNA was extracted from collected fecal samples using a commercial stool DNA extraction kit (TIANamp Stool DNA Kit) according to the manufacturer’s instructions. DNA concentration was quantified using a Qubit 2.0 fluorometer, and DNA quality and integrity were assessed by agarose gel electrophoresis prior to library preparation. Shotgun metagenomic libraries were constructed from qualified DNA samples fragmented to approximately 350 bp using a Covaris ultrasonicator, followed by end repair, A-tailing, adapter ligation, purification, and PCR amplification. After library construction, libraries were quantified using Qubit 2.0, insert size was assessed using an Agilent 2100 Bioanalyzer, and effective library concentration was determined by qPCR. Qualified libraries were pooled and sequenced on the Illumina NovaSeq X Plus platform using a paired-end 150-bp strategy. Raw reads were subjected to quality control using KneadData, including adapter trimming and removal of low-quality bases. Host-derived reads were removed by aligning sequences to the human reference genome using Bowtie2, and only high-quality non-host reads were retained for downstream analyses. Taxonomic annotation was performed using Kraken2 against a reference database based on NCBI RefSeq genomes, and relative abundances at the species and genus levels were estimated using Bracken. For paired before and after treatment samples, differential abundance analysis was performed using the Wilcoxon signed-rank test. Within-subject change values (Δ, post-treatment minus pre-treatment) were calculated, and associations between Δ*T. sanguinis* abundance and Δ clinical parameters were evaluated using Spearman’s rank correlation with Benjamini–Hochberg false discovery rate (FDR) correction.

### Statistical analysis

Continuous data are presented as mean ± SD. Statistical analyses (GraphPad Prism 9) included unpaired *t*-tests, Mann-Whitney test, paired *t*-tests, or Wilcoxon signed-rank test for two-group comparisons, one-way ANOVA with Tukey’s test for multi-group comparisons, Spearman’s test, and Benjamini–Hochberg FDR method for correlations. A *P* value below 0.05 defines statistical significance.

## RESULTS

### Prioritization of *T. sanguinis* from *Turicibacter* taxa in MASH

**Fig 1:**
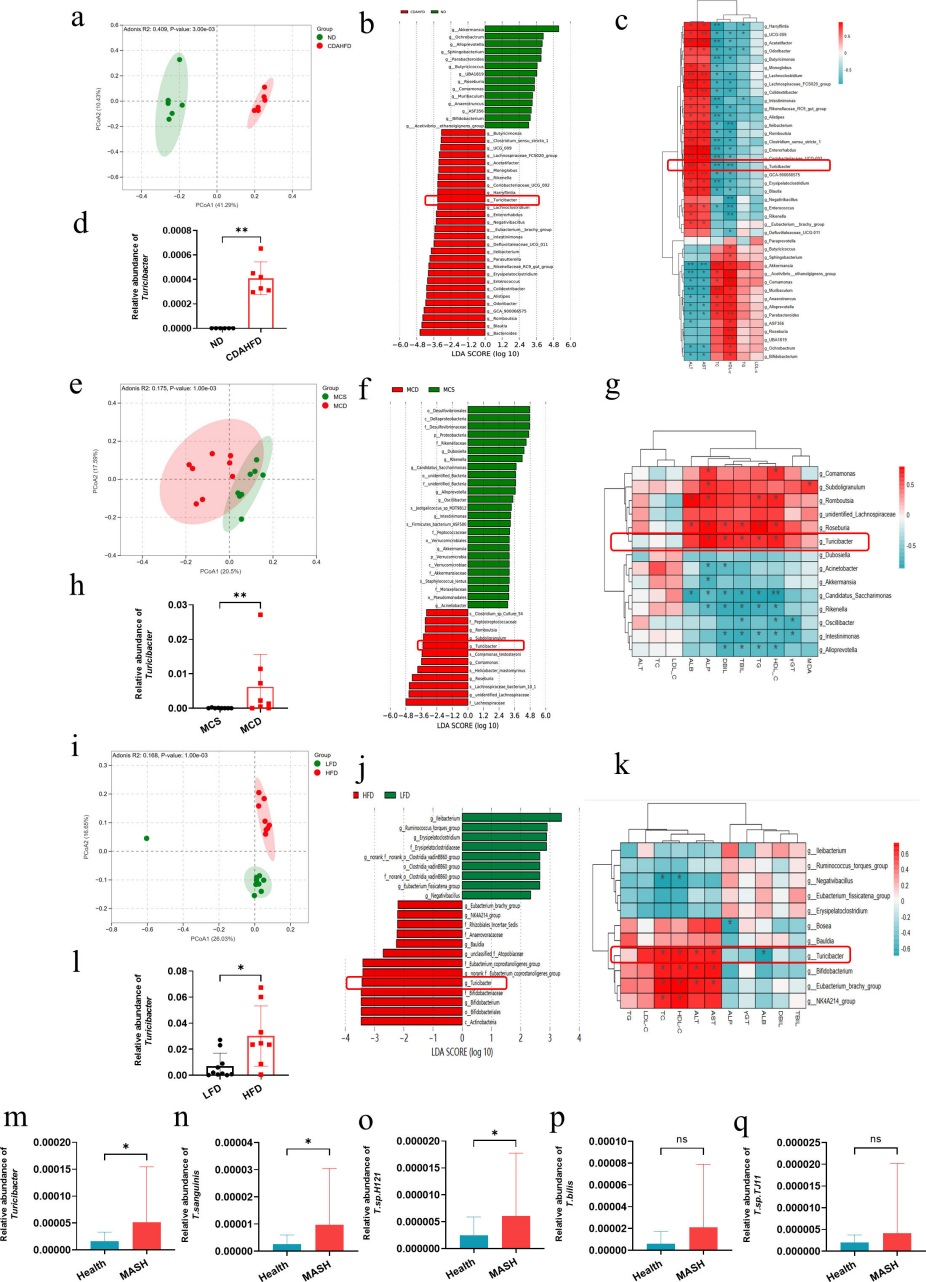
The gut microbiota alteration in MASH model and public clinical metagenomic databases. (**a**) The PCoA of CDAHFD-induced MASH and control group; *n* = 6. (**b**) The LEfSe of CDAHFD-induced MASH and control group. (**c**) The correlation between gut microbiota abundance and biochemical indicators in MASH models induced by CDAHFD. (**d**) The relative abundance of *Turicibacter* in CDAHFD-induced MASH and their control group. (**e**) The PCoA of MCD-induced MASH and control group; *n* = 8. (**f**) The LEfSe of MCD-induced MASH and control group. (**g**) The correlation between gut microbiota abundance and biochemical indicators in MASH models induced by MCD. (**h**) The relative abundance of *Turicibacter* in MCD-induced MASH and their control group. (**i**) The PCoA of HFD-induced MASH and control group; *n* = 8. (**j**) The LEfSe of HFD-induced MASH and control group. (**k**) The correlation between gut microbiota abundance and biochemical indicators in MASH models induced by HFD. (**l**) The relative abundance of *Turicibacter* in HFD-induced MASH and their control group. (**m**) The relative abundance of *Turicibacter* in MASH and healthy people; healthy: *n* = 16; MASH: *n* = 109. (**n–q**) The relative abundance of different species under the genus *Turicibacter* in MASH patients and healthy people. The data are presented as mean ± SD. Differences in data were calculated by unpaired *t*-test or Mann–Whitney test. Correlation analyses were performed using Spearman’s rank correlation. *P* values were adjusted for multiple comparisons using the Benjamini–Hochberg FDR method, and the *P* values displayed in the figures represent FDR-adjusted *P* values. *P < 0.05, **P < 0.01, ***P < 0.001 vs ND, MCS, LFD, or healthy group. ns, not significant.

To characterize gut microbial alterations in MASH, fecal samples from control and CDAHFD-induced MASH mice underwent 16S rRNA sequencing. Successful establishment of the CDAHFD-induced MASH model was confirmed prior to microbiome analysis (Fig. S2). The gut microbial community structure was distinct in MASH mice, as evidenced by distinct clustering in principal coordinate analysis (PCoA) (Fig. 1a). Among the differentially abundant genera identified by LEfSe analysis, *Turicibacter* stood out with its notable enrichment in MASH mice (Fig. 1b). To assess the generalizability of this observation, two additional MASH models, MCD-induced MASH model and HFD-induced MASH model, were also established, and successful model induction in both was confirmed before analysis (Fig. S2). Consistent alterations in gut microbiota composition were observed across these models (Fig. 1e, f, i, and j). Remarkably, *Turicibacter* was the only genus among the differential taxa identified under our analytic criteria that was consistently elevated across all three models, and the Mann-Whitney test confirmed its significant enrichment in MASH groups (Fig. 1d, h, and l). Furthermore, *Turicibacter* abundance correlated positively with liver injury markers (Fig. 1c, g, and k), suggesting a link with disease severity.

To assess the clinical relevance of these findings, the gut microbiota of healthy controls and MASH patients were compared using publicly available metagenomic data from BioProject PRJEB55534. Consistent microbiome profiling analyses were performed for this human subset, including PCoA and differential abundance analysis between the Healthy and MASH groups (Fig. S3). The results showed that *Turicibacter* was significantly enriched in MASH patients, contrasted with healthy individuals (Fig. 1m). To further pinpoint the species within this genus that might play a dominant role, the relative abundance of four *Turicibacter* taxa was analyzed, which revealed that *T. sanguinis* and *Turicibacter* sp. H121 were markedly elevated in MASH patients (Fig. 1n through q). As *Turicibacter* sp. H121 is currently annotated as an unclassified *Turicibacter* strain, despite its high genomic similarity to *Turicibacter bilis*, this study selected the well-characterized species *T. sanguinis* for subsequent functional studies.

Collectively, these multi-model and clinical metagenomic data demonstrate that *Turicibacter* is a consistently enriched genus in both MASH animals and patients. Coupled with its positive correlation with disease severity, these findings support *Turicibacter* as a candidate microbial biomarker. Future studies will focus on *T. sanguinis* to elucidate its functional mechanisms and evaluate its association with clinical therapeutic effects.

### *T. sanguinis* exacerbates MASH progression by synergistically aggravating steatosis, inflammation, and fibrosis

#### Gut microbiota influences the development of MASH

**Fig 2:**
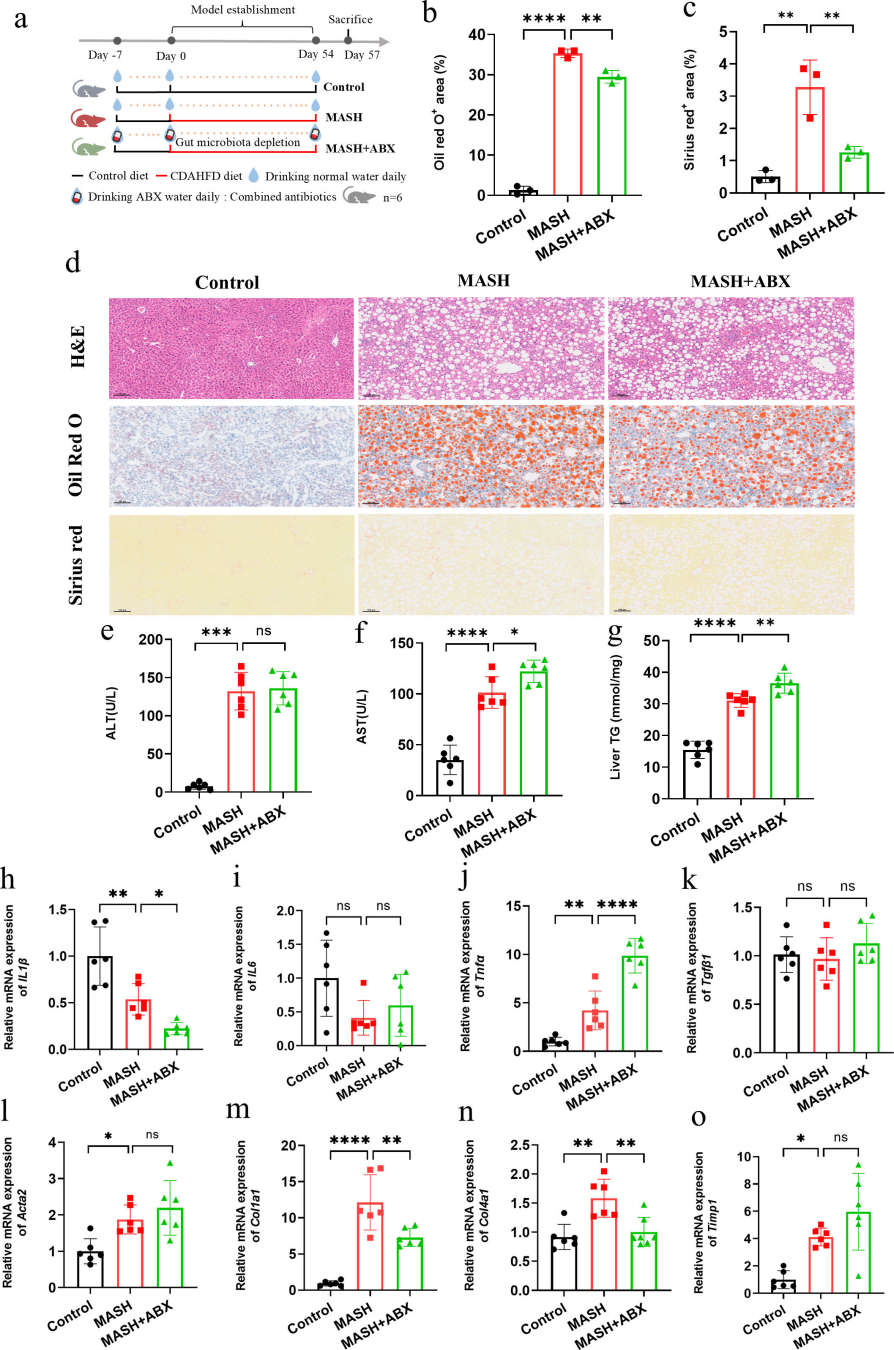
Gut microbiota influences the development of MASH. (**a**) Animal experimental schedule. (**b**) Semi-quantification of lipid accumulation. (**c**) Semi-quantification of collagen production. (**d**) Representative liver histology with H&E staining, Oil Red O staining, and Sirius Red staining. (**e**) Serum ALT level. (**f**) Serum AST level. (**g**) Liver TG level. (**h–o**) The mRNA expression of inflammation and fibrosis indices: *IL1β, IL6, Tnfα, Tgfβ1, Acta2, Col1a1, Col4a1, Timp1*. The data are presented as mean ± SD, *n* = 6; differences in data were calculated by ordinary one-way ANOVA test or Brown-Forsythe and Welch ANOVA test. *P < 0.05, **P < 0.01, ***P < 0.001, ****P < 0.0001 vs MASH group. ns, not significant.

To investigate the role of gut microbiota in MASH, a standardized antibiotic cocktail (ABX) (28) was administered to the MASH model to alter the abundance of gut microbiota (Fig. 2a). There were no significant differences in daily food intake among the groups (Fig. S4a). First, fecal 16S rRNA sequencing confirmed effective microbiota depletion, as evidenced by reduced α-diversity, marked separation in β-diversity among groups, and broad alterations in the relative abundance of gut microbial taxa (Fig. S5). Second, the ABX treatment significantly improved liver histopathological phenotypes, as evidenced by reduced lipid accumulation (Fig. 2b and d) and decreased collagen deposition (Fig. 2c and d). However, serum AST and hepatic TG levels were concurrently increased (Fig. 2f through g). At the transcriptional level, mRNA expression of key inflammatory and fibrogenic genes (*IL1β, Col1a1,* and *Col4a1*) was markedly downregulated in the ABX-treated group, whereas *Tnfα* expression showed a significant increase (Fig. 2h through o). These results indicate that the gut microbiota exerts a complex influence on the progression of MASH, with varying degrees and directions of impact on different pathological indicators.

#### Increased abundance of *T. sanguinis* exacerbates MASH progression

**Fig 3:**
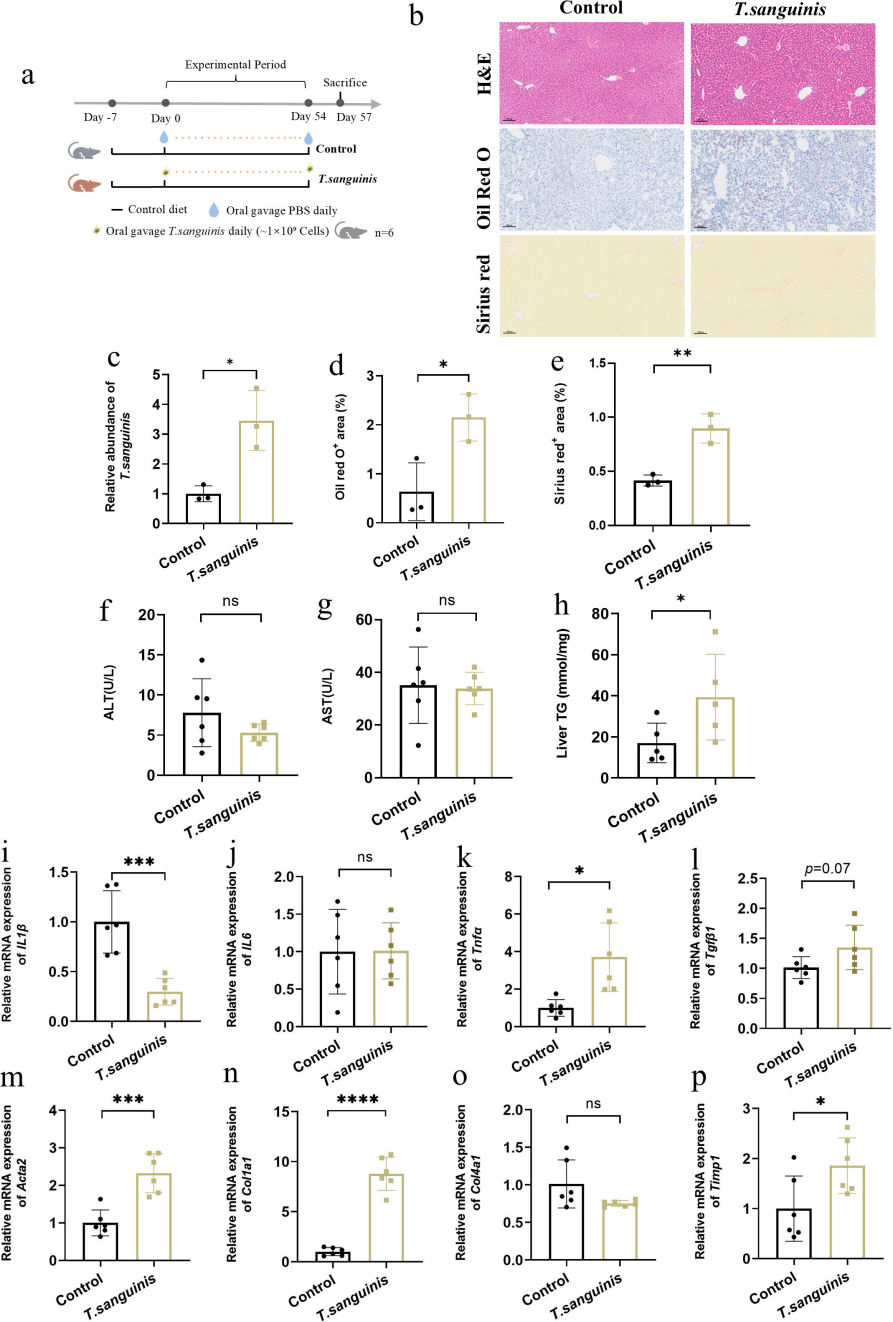
*T. sanguinis* promotes hepatic inflammation and fibrosis in normal mice. (**a**) Animal experimental schedule. (**b**) Representative liver histology with H&E staining, Oil Red O staining, and Sirius Red staining. (**c**) *T. sanguinis* relative abundance: fecal samples pooled from two mice/sample. (**d**) Semi-quantification of lipid accumulation. (**e**) Semi-quantification of collagen production. (**f**) ALT level. (**g**) AST level. (**h**) Liver TG level. (**i–p**) The mRNA expression of inflammation and fibrosis indices: *IL1β, IL6, Tnfα, Tgfβ1, Acta2, Col1a1, Col4a1, Timp1*. The data are presented as mean ± SD, *n* = 6; differences in data were calculated by an unpaired *t*-test. *P < 0.05, **P < 0.01, ***P < 0.001, ****P < 0.0001 vs control group. ns, not significant.

First, to investigate the intrinsic pathogenic potential of *T. sanguinis*, healthy control mice were administered this bacterium via oral gavage (Fig. 3a). There were no significant differences in daily food intake among the groups (Fig. S4b). The abundance of *T. sanguinis* was increased in fecal, cecal, and small intestinal contents compared with controls (Fig. 3c; S6a and b). The results observed mild but significant exacerbation of hepatic lipid deposition and fibrosis (Fig. 3d, e, and h). Furthermore, *T. sanguinis* significantly upregulated the expression of inflammation- and fibrosis-related genes, including *Tnfα*, *Tgfβ1*, *Acta2*, *Col1a1*, and *Timp1* (Fig. 3i through p).

**Fig 4:**
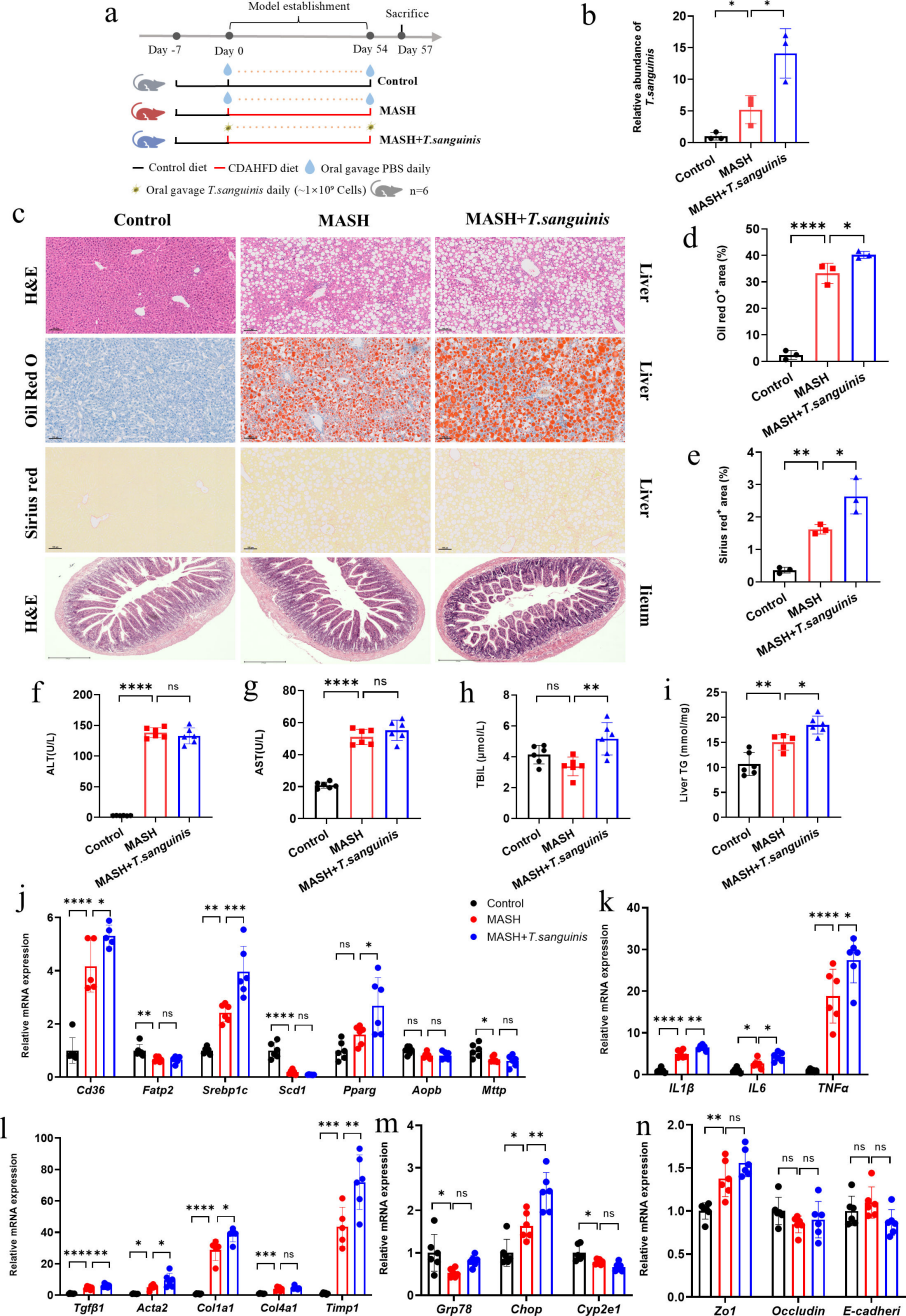
*T. sanguinis* exacerbates liver injury, steatosis, and fibrosis in MASH mice. (**a**) Animal experimental schedule. (**b**) *T. sanguinis* relative abundance: fecal samples pooled from two mice/sample, *n* = 3. (**c**) Representative liver histology with H&E staining, Oil Red O staining, and Sirius Red staining, alongside ileal with H&E staining. (**d**) Semi-quantification of lipid accumulation. (**e**) Semi-quantification of collagen production. (**f**) ALT level. (**g**) AST level. (**h**) TBIL level. (**i**) Liver TG level. (**j**) The mRNA expression of lipid uptake and synthesis index: *Cd36, Fatp2, Srebp1c, Scd1, Pparg, Aopb, Mttp*. (**k**) The mRNA expression of inflammation index: *IL1β, IL6, Tnfα*. (**l**) The mRNA expression of fibrosis index: *Tgfβ1, Acta2, Col1a1, Col4a1, Timp1*. (**m**) The mRNA expression of ER and oxidative stress index: *Grp78, Chop, Cyp2e1*. (**n**) The mRNA expression of intestinal permeability index: *Zo1, Occludin, E-cadherin*. The data are presented as mean ± SD, *n* = 6; differences in data were calculated by unpaired *t*-test, ordinary one-way ANOVA test, or Brown-Forsythe and Welch ANOVA test. *P < 0.05, **P < 0.01, ***P < 0.001, ****P < 0.0001 vs MASH group. ns, not significant.

Second, to validate whether *T. sanguinis* exacerbates disease severity on the basis of pre-existing metabolic disease, MASH mice were administered this bacterium (Fig. 4a). There were no significant differences in daily food intake among the groups (Fig. S4c). After 8 weeks of intervention, the abundance of *T. sanguinis* was also increased in feces, cecal contents, and small intestinal contents in MASH mice (Fig. 4b; Fig. S6c and d). *T. sanguinis-*treated MASH mice exhibited elevated serum TBIL (Fig. 4h), indicating aggravated liver injury. Hepatic expression of inflammatory cytokines (*IL1β, IL6, Tnfα*) was significantly elevated (Fig. 4c). Oil Red O staining and hepatic TG measurements consistently demonstrated markedly exacerbated lipid accumulation (Fig. 4c, d, and i). Genes related to lipid uptake and synthesis (*Cd36, Srebp1c, Pparg*) were also significantly upregulated (Fig. 4j). Sirius Red staining and qPCR analysis further demonstrated enhanced collagen deposition and fibrosis gene expression (*Tgfβ1, Acta2, Col1a1*, and *Timp1*) (Fig. 4c, e, and l). Additionally, endoplasmic reticulum (ER) stress marker *Chop* was elevated (Fig. 4m). Assessment of gut barrier function revealed that *T. sanguinis* had no adverse effect on its integrity in the context of MASH (Fig. 4c and n), indicating that its pathogenic effects are not primarily mediated by altering the gut’s physical structure but rather through other molecular mechanisms.

Together, these results demonstrate that *T. sanguinis* is sufficient to exacerbate MASH progression through synergistic aggravation of steatosis, inflammation, fibrosis, and ER stress. Its pathogenic effects are markedly enhanced in pre-existing liver disease, highlighting its role as an accelerant of MASH in a susceptible host environment.

### Investigation into mechanism of *T. sanguinis* pathogenicity

#### *T. sanguinis* regulates bile acid in MASH

**Fig 5:**
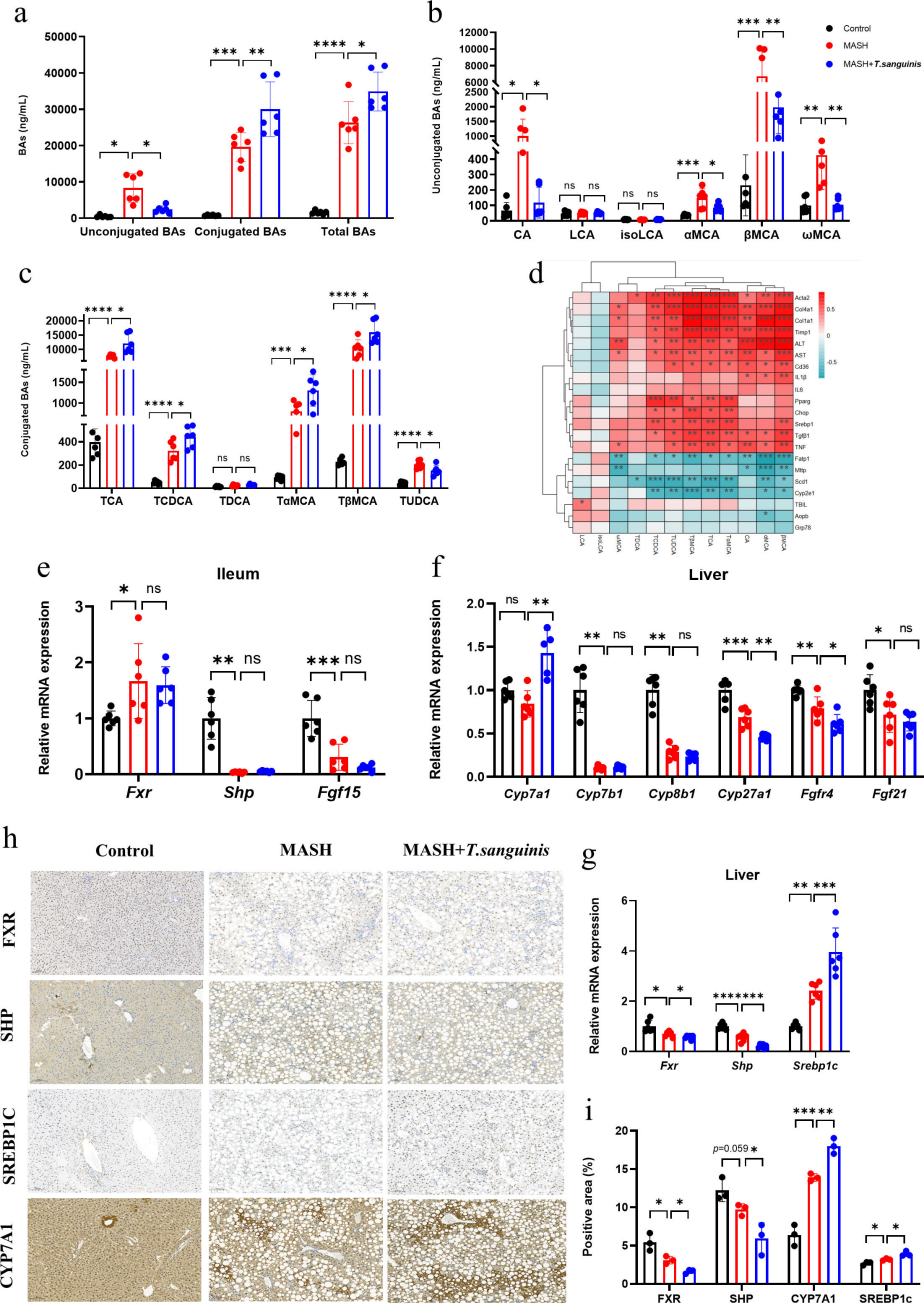
*T. sanguinis* promotes bile acid synthesis in MASH and inhibits hepatic FXR signaling. (**a**) The content of conjugated bile acids, unconjugated bile acids, and total bile acids. (**b**) The content of specific unconjugated bile acids. (**c**) The content of specific conjugated bile acids. (**d**) The correlation analysis between phenotypic indexes and bile acid levels. (**e**) The mRNA expression of *Fxr, Shp,* and *Fgf15* in ileum. (**f–g**) The mRNA expression of *Fxr* and its downstream targets in liver: *Fxr, Shp, Cyp7a1, Srebp1c, Cyp7b1, Cyp8b1, Cyp27a1, Fgfr4, Fgf21*. (**h**) Representative liver histology with immunohistochemistry. (**i**) Semi-quantification of positive area. The data are presented as mean ± SD, *n* = 6; differences in data were calculated by ordinary one-way ANOVA test or Brown-Forsythe and Welch ANOVA test. *P < 0.05, **P < 0.01, ***P < 0.001, ****P < 0.0001 vs MASH group. ns, not significant.

Previous studies have indicated that *T. sanguinis* has the ability to regulate the bile acid (29). Based on this finding, bacterial-induced alterations in bile acid composition were further investigated in MASH mice. Following intragastric administration of *T. sanguinis*, an elevated pool of total and conjugated bile acids, with a concurrent reduction in unconjugated species (Fig. 5a). Specifically, among the unconjugated bile acids, *T. sanguinis* treatment significantly reduced cholic acid (CA), α-muricholic acid (αMCA), β-muricholic acid (βMCA), and ω-muricholic acid (ωMCA) (Fig. 5b). In contrast, among the conjugated bile acids, the levels of taurocholic acid (TCA), taurochenodeoxycholic acid (TCDCA), tauro-α-muricholic acid (TαMCA), tauro-β-muricholic acid (TβMCA) were all significantly increased (Fig. 5c). Further correlation analysis revealed that most liver phenotypic indicators were positively correlated with conjugated bile acids, particularly TCA, TαMCA, and TβMCA (Fig. 5d). These findings suggest that *T. sanguinis* may exacerbate MASH progression by regulating the bile acid metabolic profile.

#### *T. sanguinis* inhibited hepatic FXR signaling in MASH

Given the central role of FXR in MASH, this study assessed the expression of this intestine/liver-enriched bile acid receptor and its downstream gene network. Compared with the control group, *T. sanguinis* treatment exerted a tissue-specific effect, significantly suppressing hepatic *Fxr* expression while leaving intestinal levels unaltered. Further analysis revealed the expression of *Fxr* downstream target genes. In the liver, *T. sanguinis* treatment inhibited the expression of *Shp*, *Cyp27a1*, and *Fgfr4*, with a concurrent upregulation of *Cyp7a1* and *Srebp1c*, whereas intestinal *Shp* and *Fgf15* expression remained unaltered (Fig. 5e through g). Integrating these results, *T. sanguinis* likely exerts its effects by inhibiting hepatic *Fxr* and *Shp* while promoting the expression of *Cyp7a1* and *Srebp1c*. In addition, immunohistochemical detection consistently showed similar trends at the protein level for FXR, SHP, CYP7A1, and SREBP1c (Fig. 5h and i). These results imply that *T. sanguinis* inhibited hepatic FXR signaling, enhanced bile acid synthesis, and promoted lipogenesis.

### Decreased *T. sanguinis* abundance in MASH patients after treatment

**Fig 6:**
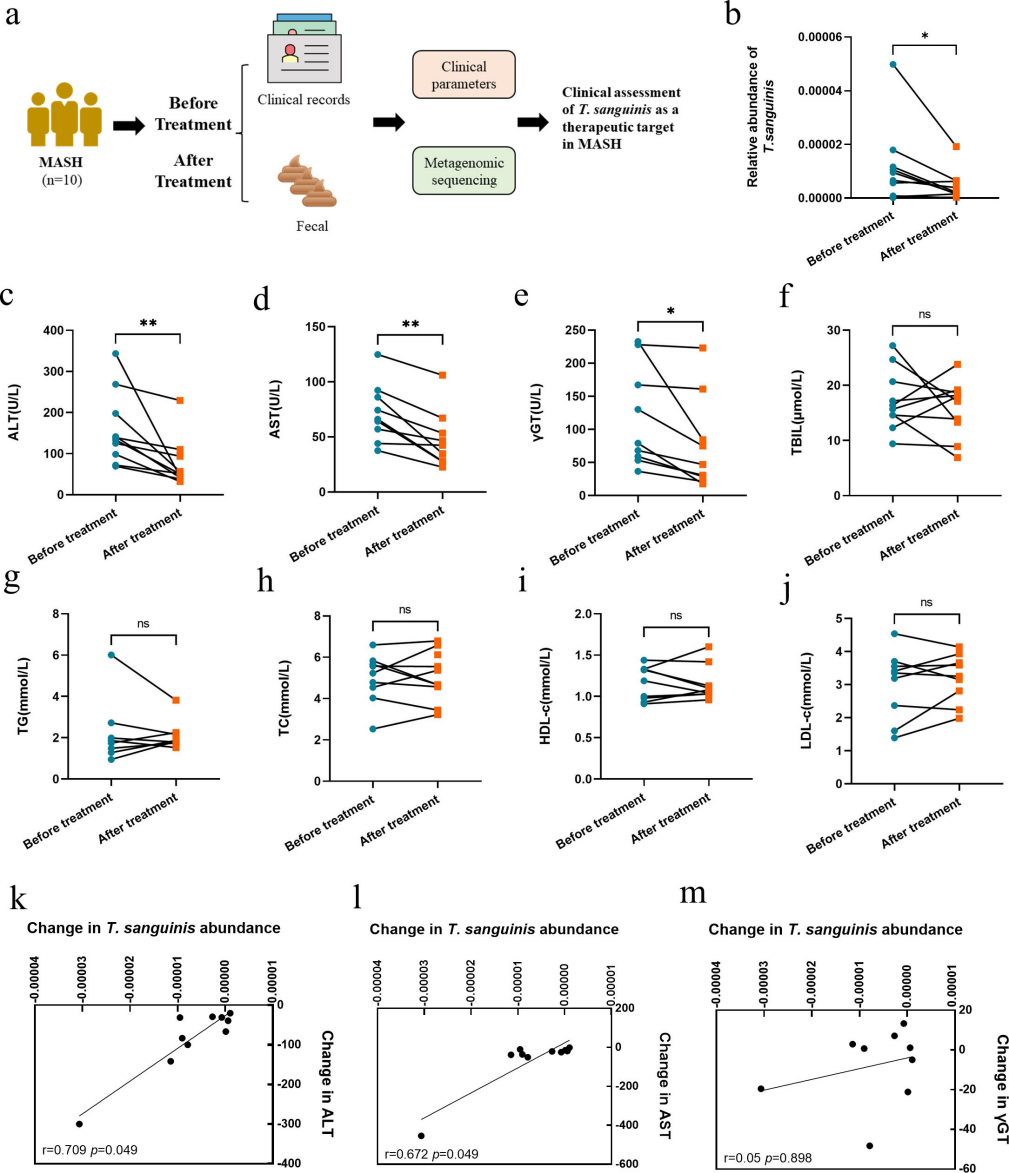
*T. sanguinis* abundance decreases after treatment and correlates with liver injury markers in MASH patients. (**a**) Clinical sample collection flowchart. (**b**) Relative abundance of *T. sanguinis* in MASH patients and treated MASH patients. (**c–j**) Liver injury and blood lipid indicators: ALT, AST, TBIL, γ-GT, TG, TC, HDL-c, LDL-c. (**k–m**) Correlation between the change of relative abundance of *T. sanguinis* and the change of ALT, AST, and γ-GT. The data are presented as mean ± SD; *n* = 10; differences in data were calculated by paired *t*-test or Wilcoxon signed-rank test. Correlation analyses were performed using Spearman’s rank correlation. *P* values were adjusted for multiple comparisons using the Benjamini–Hochberg FDR method, and the *P* values displayed in the figures represent FDR-adjusted *P* values. *P < 0.05, **P < 0.01 vs before treatment group; ns, not significant.

To evaluate the relationship between *T. sanguinis* and the therapeutic responses in MASH, paired fecal samples were obtained from 10 MASH patients before and after a course of standard clinical treatment with silibinin and vitamin E (Fig. 6a). Clinical assessments demonstrated significant improvements in liver function parameters, such as ALT, AST, and γGT, following pharmacotherapy (Fig. 6c through j). Consistent with these improvements, the relative abundance of *T. sanguinis* was significantly reduced after treatment compared with before (Fig. 6b). Other genus and species that changed following treatment are shown in Fig. S7a through c. To avoid potential concerns related to the non-independence of paired samples, correlation analyses were performed using the change (Δ) values for each individual (after treatment minus before treatment). When correlations between the changes in all differential taxa and clinical indicators were assessed and corrected for multiple testing using the Benjamini–Hochberg FDR method, no associations remained statistically significant, likely due to the limited sample size and the large multiple-testing burden (Fig. S7d). Given that *T. sanguinis* was identified as the key taxon in this study, a hypothesis-driven correlation analysis was performed, focusing specifically on the relationship between the change in *T. sanguinis* abundance and the changes in liver biochemical parameters. Δ*T. sanguinis* abundance was significantly correlated with ΔALT and ΔAST after FDR correction (Fig. 6k through m). These results suggest that treatment-associated reductions in *T. sanguinis* are associated with improvements in liver injury markers.

Collectively, these findings indicate that *T. sanguinis* abundance is modifiable during standard pharmacotherapy and is associated with changes in clinical liver injury indices, supporting its potential relevance as a microbiota-linked indicator of treatment response in MASH.

## DISCUSSION

In the present study, *Turicibacter sanguinis* emerged as a candidate gut microbial driver of MASH progression and was shown to aggravate disease phenotypes *in vivo*, with evidence suggesting involvement of bile acid alterations and associated modulation of hepatic FXR signaling. By integrating initial screening in CDAHFD-induced MASH model, with independent validation in other MASH models, supplemented by public clinical metagenomic data and functional assays, this study provides convergent evidence that *T. sanguinis* not only correlates with but functionally contributes to MASH pathogenesis by synergistically exacerbating hepatic steatosis, inflammation, and fibrosis. Its abundance consistently reflects disease severity across preclinical models and human patients, and further functional investigations confirmed that *T. sanguinis* enrichment markedly amplifies disease phenotypes under metabolic stress. Although previous studies have reported associations between the genus *Turicibacter* and metabolic liver disease, they were limited to genus-level observations. For example, Yi Wu et al. reported a notable increase in *Turicibacter* in HFD-induced MASLD mouse models (30), and Cristina Rodriguez-Diaz et al. further revealed a correlation between *Turicibacter* abundance and disease severity: MASLD patients with significant liver fibrosis had markedly higher *Turicibacter* levels compared to those with no/mild fibrosis (31). However, these studies did not identify specific causative species nor perform functional validation. Similarly, Lynch et al. provide pivotal insights into the strain-specific bile acid modifications and lipid metabolic influences mediated by *Turicibacter* species (29), their work primarily emphasized bacterial genetics and did not fully elucidate the functional role of this bacterium in the context of specific human diseases, such as MASH. In this regard, this study extends prior observations by providing species-level and functional evidence supporting a contributory role for *T. sanguinis* in MASH progression under our experimental conditions. At the same time, an important limitation should be acknowledged: we did not perform comparative colonization experiments using other *Turicibacter* species or strains, nor did we compare *T. sanguinis* with other bile acid-modifying commensals. Therefore, while our data indicate that *T. sanguinis* is sufficient to worsen MASH phenotypes and engage a bile acid–FXR axis, they do not support the conclusion that this effect is exclusive to this species. Future studies comparing multiple *Turicibacter* isolates and relevant control bacteria will be necessary to define the breadth and strain specificity of this effect.

In order to bolster the rigor and clinical translatability of our evidence, this study adopted an integrated multi-model strategy, utilizing CDAHFD-, MCD-, and HFD-induced MASH models in combination with clinical metagenomic data validation. The use of multiple models helps avoid dietary or pathophysiological biases inherent in single-model approaches, as each model captures distinct aspects of human MASH: the MCD model rapidly induces inflammation and fibrosis but is accompanied by significant weight loss; the HFD model better mimics metabolic syndrome but requires a longer period to develop fibrosis and exhibits high individual variability; whereas the CDAHFD model comprehensively recapitulates key pathologies of MASH, including severe steatosis, ballooning, prominent inflammation, and significant fibrosis, within a relatively short timeframe with high reproducibility. Therefore, the CDAHFD model was selected for subsequent functional validation and mechanistic investigations. Through cross-model validation, this study identified a consistently enriched microbe across both animal models and clinical samples, indicating that its association with MASH is model-independent. This strategy helps distinguish truly pathologically relevant microbes from model-specific signals, thereby enhancing the biological reliability of the findings, similar to the multi-model approach used for biomarker validation in fields such as oncology and immunology. However, heterogeneity among models may obscure certain context-dependent microbial contributions, requiring careful interpretation of differentially abundant taxa. Despite this limitation, the consistency between multi-model data and clinical findings significantly strengthens the credibility of the core discoveries. Future studies further validating the pathogenic role of *T. sanguinis* in long-term HFD or Western MASH models would help improve the generalizability and reliability of the conclusions drawn in this study.

This study also suggested that, in the CDAHFD model, antibiotic treatment should not be simply regarded as exerting a uniformly protective effect on MASH, but rather understood as a complex process of gut microbiota remodeling. Although antibiotic exposure alleviated certain features of steatosis and fibrosis, it was also accompanied by elevated serum AST and TNFα levels, indicating increased hepatocellular injury and inflammatory activation. These observations suggest that antibiotic intervention may exert dissociated effects across different pathological dimensions of MASH rather than producing a uniformly beneficial outcome. One possible explanation is that antibiotics not only suppress potentially pathogenic bacteria but also eliminate beneficial commensals that are important for maintaining intestinal microbial balance and immune homeostasis. As a result, antibiotic treatment may generate a residual microbiota characterized by reduced diversity and functional disequilibrium, rather than a biologically neutral depleted state. Consistent with this interpretation, we observed an expansion of several opportunistic or pathogen-associated genera after antibiotic treatment, including *Morganella, Escherichia-Shigella, Klebsiella, Enterobacter, Comamonas*, and *Proteus*. Previous studies have shown that antibiotic exposure can promote abnormal intestinal colonization by *Klebsiella*, and that high-alcohol-producing *Klebsiella pneumoniae* can directly induce fatty liver and accelerate MASLD progression (32, 33). Therefore, enrichment of such opportunistic taxa in the residual microbiota may help explain the dissociated hepatic phenotype observed in this study, in which lipid deposition and collagen accumulation were reduced, whereas inflammatory injury was aggravated. In addition, the perturbing effect of antibiotics on the gut microbiota is highly dependent on dosage, duration of intervention, route of administration, and the host’s baseline microbial composition (28, 34). Thus, even when the same antibiotic cocktail and CDAHFD model are used, different experimental conditions may still result in markedly different residual microbial communities, which may in turn lead to distinct hepatic phenotypes. This may be one of the reasons why our findings are not entirely consistent with previous reports (35).

A preliminary mechanistic exploration suggested that *T. sanguinis* may aggravate MASH progression by reshaping bile acid metabolism. *T. sanguinis* regulated the bile acid profiles by increasing conjugated bile acids, including TCA, TCDCA, TαMCA, TβMCA, and decreasing unconjugated species, such as CA, αMCA, βMCA, and ωMCA. These altered profiles likely inhibit hepatic FXR signaling, resulting in suppressed SHP and elevated CYP7A1 and SREBP1c expression, promoting bile acid synthesis and lipid accumulation. Importantly, intestinal FXR signaling remained unchanged, suggesting *T. sanguinis* influences liver pathology through direct modulation of portal vein bile acid composition. Although microbial bile acid biotransformation primarily occurs within the intestinal lumen, intestinal and hepatic FXR are exposed to distinct bile acid microenvironments. Active ileal bile acid reabsorption depends on apical uptake via ASBT, followed by basolateral export through OSTα/β into the portal circulation. During this process, absorbed bile acids activate FXR within ileal enterocytes and induce target genes, such as FGF15/19, thereby reflecting signaling driven by local luminal bile acid exposure. In contrast, hepatocytes efficiently extract portal vein bile acids via transporters, such as NTCP, enabling hepatic FXR to more directly integrate changes in the composition and load of bile acids reaching the liver. Therefore, if gut microbes exhibit region-specific bile acid transformation capacities, they may selectively reshape the bile acid pool delivered to the liver by altering the composition of bile acids entering the portal circulation. Under such conditions, hepatic FXR activity could be preferentially affected, even in the absence of substantial changes in overall luminal bile acid exposure in the ileum (36, 37). Functionally, the increased conjugated bile acids observed may further contribute to liver pathology. For instance, TCDCA promotes pyroptosis and inflammation (38), while TCA activates hepatic stellate cells via TLR4 signaling (39). These observations provide a plausible framework for understanding how *T. sanguinis*-associated bile acid remodeling may aggravate MASH, although direct mechanistic validation, including rescue experiments, will be required in future studies.

Recent studies have highlighted substantial functional heterogeneity within the *Turicibacter* genus. Lynch et al. demonstrated that different strains possess distinct bile-modifying capacities due to variation in bile salt hydrolases and hydroxysteroid dehydrogenases, resulting in strain-dependent effects on bile acid conjugation status (29). In addition to their metabolic activity, *Turicibacter* species also exhibit close interactions with host bile acid regulatory pathways. Kemis et al. reported that the abundance of *Turicibacter* is closely associated with host bile acid transport and enterohepatic circulation, particularly through its regulation of the ileal transporter SLC10A2. These findings suggest that *Turicibacter* may function not only as a modulator of bile acids but also as a responder to host mechanisms that maintain bile acid homeostasis (40). Such bidirectional microbiota–host interactions may be particularly relevant in the context of MASH, a disease characterized by profound disturbances in bile acid synthesis, conjugation dynamics, and enterohepatic circulation. Under these pathological conditions, host metabolism shifts toward enhanced taurine conjugation and bile acid transport. This altered metabolic environment may reshape the functional role of *Turicibacter*, potentially transforming it from its classical “deconjugating” role observed in healthy or germ-free models into one that contributes to the accumulation of conjugated bile acids. This disease-context-dependent interaction between the microbiota and host bile acid metabolism may partly explain the differences between the bile acid profiles observed in our study and those reported previously. Furthermore, Fung et al. revealed that *T. sanguinis* can sense host 5-HT through its serotonin transporter, thereby regulating sporulation, colonization fitness, and host lipid metabolism (41). Together, these findings suggest that *T. sanguinis* may influence host metabolic homeostasis through integrated interactions involving bile acid metabolism and host signaling pathways. Accordingly, our findings broaden the current understanding of the pathological potential of *Turicibacter* species and support further investigation of *T. sanguinis* as a candidate microbiota-associated therapeutic target in MASH.

These findings may also have profound clinical implications. Analysis revealed that the abundance of *T. sanguinis* decreased following standardized pharmacotherapy, and the magnitude of its reduction was positively correlated with improvements in liver injury markers, including ALT and AST. In the microbiome-wide analysis, no significant correlations remained after FDR correction, likely reflecting the limited statistical power of the paired cohort and the large multiple-testing burden inherent to microbiome studies. In contrast, the hypothesis-driven analysis focusing on *T. sanguinis,* a taxon with mechanistic relevance supported by the present study, revealed significant associations with improvements in liver biochemical parameters. Together, these observations suggest that *T. sanguinis* may represent a candidate noninvasive biomarker for treatment response and highlight its potential relevance as a microbiota-associated therapeutic target. To further validate these findings, an ongoing expanded prospective study will further evaluate the predictive value of *T. sanguinis* abundance for treatment outcomes. Additionally, given the correlation between *T. sanguinis* abundance and FXR signaling, it will be of interest in future studies to determine whether baseline *T. sanguinis* abundance is related to responsiveness to FXR-targeting therapies, such as obeticholic acid. More broadly, the results open possibilities for precisely targeting *T. sanguinis* through antimicrobials, phage-based therapies, or dietary interventions without broadly disrupting commensal microbes. However, such translational applications remain speculative at present and will require rigorous validation in future studies.

In conclusion, this study advances the field by moving beyond genus-level association to establish *T. sanguinis* as a functionally validated, clinically relevant pathogen in MASH and provides a comprehensive mechanistic framework linking its abundance to bile acid dysregulation, impaired FXR signaling, and exacerbated liver pathology. These findings provide new insights into the gut-liver axis in metabolic diseases and provide a foundation for developing microbiome-based diagnostics and targeted therapies. Future research should explore specific interventions against *T. sanguinis* and investigate whether reducing its abundance can ameliorate or reverse MASH in humans.

### Conclusion

This study prioritizes *Turicibacter sanguinis* as a clinically relevant candidate associated with MASH and demonstrates that its presence is sufficient to exacerbate MASH phenotypes, accompanied by bile acid dysregulation and suppression of hepatic FXR signaling. Its reduction after successful treatment supports its potential as a modifiable microbial marker and motivates future work to test whether targeted reduction of *T. sanguinis* can confer therapeutic benefit.

## Data Availability

All data supporting the findings of this study are available within the article or from the corresponding author upon reasonable request. The 16S rRNA sequencing data have been deposited in the NCBI under accession number PRJNA1245296. The human metagenomic sequencing data have been deposited in the NCBI under accession number PRJNA1431906. The completed STORMS checklist has been uploaded to the Open Science Framework (OSF) and is publicly available as part of the study materials: https://osf.io/5z3pj/overview?view_only=5231470df4534aac976dd1640331c624.

## SUPPLEMENTAL MATERIAL

Supplemental figures msystems.00292-26-s0001.docx Fig. S1 to S7.

Supplemental tables msystems.00292-26-s0002.xlsx Tables S1 to S4.

ASM does not own the copyrights to Supplemental Material that may be linked to, or accessed through, an article. The authors have granted ASM a non-exclusive, world-wide license to publish the Supplemental Material files. Please contact the corresponding author directly for reuse.

## References

[1] Fu J-T, Liu J, Wu W-B, Chen Y-T, Lu G-D, Cao Q, Meng H-B, Tong J, Zhu J-H, Wang X-J, Liu Y, Zhuang C, Sheng C, Shen F-M, Liu X, Wang H, Yu Y, Zhang Y, Liang H-Y, Zhang J-B, Li D-J, Li X, Wang Z-B, Wang P. 2024. Targeting EFHD2 inhibits interferon-γ signaling and ameliorates non-alcoholic steatohepatitis. J Hepatol 81:389–403. doi:10.1016/j.jhep.2024.04.009 38670321

[2] Younossi ZM, Tampi RP, Racila A, Qiu Y, Burns L, Younossi I, Nader F. 2020. Economic and clinical burden of nonalcoholic steatohepatitis in patients with type 2 diabetes in the U.S. Diabetes Care 43:283–289. doi:10.2337/dc19-1113 31658974

[3] Targher G, Byrne CD, Tilg H. 2024. MASLD: a systemic metabolic disorder with cardiovascular and malignant complications. Gut 73:691–702. doi:10.1136/gutjnl-2023-330595 38228377

[4] Sheka AC, Adeyi O, Thompson J, Hameed B, Crawford PA, Ikramuddin S. 2020. Nonalcoholic steatohepatitis: a review. JAMA 323:1175–1183. doi:10.1001/jama.2020.2298 32207804

[5] Zhang X, Lau H-H, Yu J. 2025. Pharmacological treatment for metabolic dysfunction-associated steatotic liver disease and related disorders: current and emerging therapeutic options. Pharmacol Rev 77:100018. doi:10.1016/j.pharmr.2024.100018 40148030

[6] Harrison SA, Bedossa P, Guy CD, Schattenberg JM, Loomba R, Taub R, Labriola D, Moussa SE, Neff GW, Rinella ME, et al.. 2024. A phase 3, randomized, controlled trial of resmetirom in NASH with liver fibrosis. N Engl J Med 390:497–509. doi:10.1056/NEJMoa2309000 38324483

[7] Xu X, Poulsen KL, Wu L, Liu S, Miyata T, Song Q, Wei Q, Zhao C, Lin C, Yang J. 2022. Targeted therapeutics and novel signaling pathways in non-alcohol-associated fatty liver/steatohepatitis (NAFL/NASH). Signal Transduct Target Ther 7:287. doi:10.1038/s41392-022-01119-3 35963848 PMC9376100

[8] Tilg H, Adolph TE, Trauner M. 2022. Gut-liver axis: pathophysiological concepts and clinical implications. Cell Metab 34:1700–1718. doi:10.1016/j.cmet.2022.09.017 36208625

[9] Zeevi D, Korem T, Godneva A, Bar N, Kurilshikov A, Lotan-Pompan M, Weinberger A, Fu J, Wijmenga C, Zhernakova A, Segal E. 2019. Structural variation in the gut microbiome associates with host health. Nature 568:43–48. doi:10.1038/s41586-019-1065-y 30918406

[10] de Vos WM, Tilg H, Van Hul M, Cani PD. 2022. Gut microbiome and health: mechanistic insights. Gut 71:1020–1032. doi:10.1136/gutjnl-2021-326789 35105664 PMC8995832

[11] Wu J, Wang K, Wang X, Pang Y, Jiang C. 2021. The role of the gut microbiome and its metabolites in metabolic diseases. Protein Cell 12:360–373. doi:10.1007/s13238-020-00814-7 33346905 PMC8106557

[12] Vallianou N, Christodoulatos GS, Karampela I, Tsilingiris D, Magkos F, Stratigou T, Kounatidis D, Dalamaga M. 2021. Understanding the role of the gut microbiome and microbial metabolites in non-alcoholic fatty liver disease: current evidence and perspectives. Biomolecules 12:56. doi:10.3390/biom12010056 35053205 PMC8774162

[13] Aron-Wisnewsky J, Vigliotti C, Witjes J, Le P, Holleboom AG, Verheij J, Nieuwdorp M, Clément K. 2020. Gut microbiota and human NAFLD: disentangling microbial signatures from metabolic disorders. Nat Rev Gastroenterol Hepatol 17:279–297. doi:10.1038/s41575-020-0269-9 32152478

[14] Hsu CL, Schnabl B. 2023. The gut-liver axis and gut microbiota in health and liver disease. Nat Rev Microbiol 21:719–733. doi:10.1038/s41579-023-00904-3 37316582 PMC10794111

[15] Marra F, Svegliati-Baroni G. 2018. Lipotoxicity and the gut-liver axis in NASH pathogenesis. J Hepatol 68:280–295. doi:10.1016/j.jhep.2017.11.014 29154964

[16] Adorini L, Trauner M. 2023. FXR agonists in NASH treatment. J Hepatol 79:1317–1331. doi:10.1016/j.jhep.2023.07.034 37562746

[17] Albillos A, de Gottardi A, Rescigno M. 2020. The gut-liver axis in liver disease: pathophysiological basis for therapy. J Hepatol 72:558–577. doi:10.1016/j.jhep.2019.10.003 31622696

[18] Li H, Wang X-K, Tang M, Lei L, Li J-R, Sun H, Jiang J, Dong B, Li H-Y, Jiang J-D, Peng Z-G. 2024. Bacteroides thetaiotaomicron ameliorates mouse hepatic steatosis through regulating gut microbial composition, gut-liver folate and unsaturated fatty acids metabolism. Gut Microbes 16:2304159. doi:10.1080/19490976.2024.2304159 38277137 PMC10824146

[19] Chen B, Sun L, Zeng G, Shen Z, Wang K, Yin L, Xu F, Wang P, Ding Y, Nie Q, Wu Q, Zhang Z, Xia J, Lin J, Luo Y, Cai J, Krausz KW, Zheng R, Xue Y, Zheng M-H, Li Y, Yu C, Gonzalez FJ, Jiang C. 2022. Gut bacteria alleviate smoking-related NASH by degrading gut nicotine. Nature 610:562–568. doi:10.1038/s41586-022-05299-4 36261549 PMC9589931

[20] Yang M, Qi X, Li N, Kaifi JT, Chen S, Wheeler AA, Kimchi ET, Ericsson AC, Rector RS, Staveley-O’Carroll KF, Li G. 2023. Western diet contributes to the pathogenesis of non-alcoholic steatohepatitis in male mice via remodeling gut microbiota and increasing production of 2-oleoylglycerol. Nat Commun 14:228. doi:10.1038/s41467-023-35861-1 36646715 PMC9842745

[21] Wang J, Hu Q, Wang J, Lang L, Wei S, Li H, Jing M, Ma X, Zhao Y, Zhou X. 2025. Role of gut microbiota and fecal metabolites in the protective effect of soybean pulp-rich diet against estrogen-induced cholestasis in rats. Curr Res Food Sci 10:100990. doi:10.1016/j.crfs.2025.100990 39995468 PMC11849669

[22] Li T-T, Tong A-J, Liu Y-Y, Huang Z-R, Wan X-Z, Pan Y-Y, Jia R-B, Liu B, Chen X-H, Zhao C. 2019. Polyunsaturated fatty acids from microalgae Spirulina platensis modulates lipid metabolism disorders and gut microbiota in high-fat diet rats. Food Chem Toxicol 131:110558. doi:10.1016/j.fct.2019.06.005 31175915

[23] Bolger AM, Lohse M, Usadel B. 2014. Trimmomatic: a flexible trimmer for Illumina sequence data. Bioinformatics 30:2114–2120. doi:10.1093/bioinformatics/btu170 24695404 PMC4103590

[24] Langmead B, Salzberg SL. 2012. Fast gapped-read alignment with Bowtie 2. Nat Methods 9:357–359. doi:10.1038/nmeth.1923 22388286 PMC3322381

[25] Wood DE, Lu J, Langmead B. 2019. Improved metagenomic analysis with Kraken 2. Genome Biol 20:257. doi:10.1186/s13059-019-1891-0 31779668 PMC6883579

[26] Lu J, Breitwieser FP, Thielen P, Salzberg SL. 2017. Bracken: estimating species abundance in metagenomics data. PeerJ Comput Sci 3:e104. doi:10.7717/peerj-cs.104 PMC12016282 40271438

[27] Ma C, Yuan D, Renaud SJ, Zhou T, Yang F, Liou Y, Qiu X, Zhou L, Guo Y. 2022. Chaihu-shugan-san alleviates depression-like behavior in mice exposed to chronic unpredictable stress by altering the gut microbiota and levels of the bile acids hyocholic acid and 7-ketoDCA. Front Pharmacol 13:1040591. doi:10.3389/fphar.2022.1040591 36339629 PMC9627339

[28] Panyod S, Wu W-K, Lee Y-P, Lin T-Y, Kuo P-C, Yen C-S, Chen Y-H, Chuang H-L, Lo I-H, Yang Y-T, Lo Y-L, Liao W-T, Chen C-T, Chow L-P, Lin C-H, Lee Y-C, Hsu C-C, Wu M-S. 2026. Refining antibiotic cocktail regimens for pseudo-germ-free mice and their impact on gut microbiome and pancreatic tumor proteomics. J Adv Res 83:391–408. doi:10.1016/j.jare.2025.08.015 40816352 PMC13131506

[29] Lynch JB, Gonzalez EL, Choy K, Faull KF, Jewell T, Arellano A, Liang J, Yu KB, Paramo J, Hsiao EY. 2023. Gut microbiota Turicibacter strains differentially modify bile acids and host lipids. Nat Commun 14:3669. doi:10.1038/s41467-023-39403-7 37339963 PMC10281990

[30] Wu Y, Yin W, Hao P, Chen Y, Yu L, Yu X, Wu Y, Li X, Wang W, Zhou H, Yuan Y, Quan X, Yu Y, Hu B, Chen S, Zhou Z, Sun W. 2024. Polysaccharide from Panax japonicus C.A. Mey prevents non-alcoholic fatty liver disease development based on regulating liver metabolism and gut microbiota in mice. Int J Biol Macromol 260:129430. doi:10.1016/j.ijbiomac.2024.129430 38228199

[31] Rodriguez-Diaz C, Taminiau B, García-García A, Cueto A, Robles-Díaz M, Ortega-Alonso A, Martín-Reyes F, Daube G, Sanabria-Cabrera J, Jimenez-Perez M, Isabel Lucena M, Andrade RJ, García-Fuentes E, García-Cortes M. 2022. Microbiota diversity in nonalcoholic fatty liver disease and in drug-induced liver injury. Pharmacol Res 182:106348. doi:10.1016/j.phrs.2022.106348 35817360

[32] Kienesberger S, Cosic A, Kitsera M, Raffl S, Hiesinger M, Leitner E, Halwachs B, Gorkiewicz G, Glabonjat RA, Raber G, Lembacher-Fadum C, Breinbauer R, Schild S, Zechner EL. 2022. Enterotoxin tilimycin from gut-resident Klebsiella promotes mutational evolution and antibiotic resistance in mice. Nat Microbiol 7:1834–1848. doi:10.1038/s41564-022-01260-3 36289400 PMC9613472

[33] Yuan J, Chen C, Cui J, Lu J, Yan C, Wei X, Zhao X, Li N, Li S, Xue G, et al.. 2019. Fatty liver disease caused by high-alcohol-producing Klebsiella pneumoniae. Cell Metab 30:675–688. doi:10.1016/j.cmet.2019.08.018 31543403

[34] Zhang S, Chen DC. 2019. Facing a new challenge: the adverse effects of antibiotics on gut microbiota and host immunity. Chin Med J (Engl) 132:1135–1138. doi:10.1097/CM9.0000000000000245 30973451 PMC6511407

[35] Yi F, Wang J, Chen Y, Xia Y, Zhou H, Huang Y, Shen Y, Fang S, Wang X, Zhang Y, Chen Y, Chen D. 2025. Nicotine exacerbates MASH via inducing intestinal dysbiosis and barrier dysfunction. Commun Biol 8:1748. doi:10.1038/s42003-025-09280-5 41350926 PMC12680697

[36] Cai J, Rimal B, Jiang C, Chiang JYL, Patterson AD. 2022. Bile acid metabolism and signaling, the microbiota, and metabolic disease. Pharmacol Ther 237:108238. doi:10.1016/j.pharmthera.2022.108238 35792223

[37] Fleishman JS, Kumar S. 2024. Bile acid metabolism and signaling in health and disease: molecular mechanisms and therapeutic targets. Signal Transduct Target Ther 9:97. doi:10.1038/s41392-024-01811-6 38664391 PMC11045871

[38] Zhao Q, Dai M-Y, Huang R-Y, Duan J-Y, Zhang T, Bao W-M, Zhang J-Y, Gui S-Q, Xia S-M, Dai C-T, Tang Y-M, Gonzalez FJ, Li F. 2023. Parabacteroides distasonis ameliorates hepatic fibrosis potentially via modulating intestinal bile acid metabolism and hepatocyte pyroptosis in male mice. Nat Commun 14:1829. doi:10.1038/s41467-023-37459-z 37005411 PMC10067939

[39] Liu Z, Zhang Z, Huang M, Sun X, Liu B, Guo Q, Chang Q, Duan Z. 2018. Taurocholic acid is an active promoting factor, not just a biomarker of progression of liver cirrhosis: evidence from a human metabolomic study and in vitro experiments. BMC Gastroenterol 18:112. doi:10.1186/s12876-018-0842-7 29996772 PMC6042259

[40] Kemis JH, Linke V, Barrett KL, Boehm FJ, Traeger LL, Keller MP, Rabaglia ME, Schueler KL, Stapleton DS, Gatti DM, Churchill GA, Amador-Noguez D, Russell JD, Yandell BS, Broman KW, Coon JJ, Attie AD, Rey FE. 2019. Genetic determinants of gut microbiota composition and bile acid profiles in mice. PLoS Genet 15:e1008073. doi:10.1371/journal.pgen.1008073 31465442 PMC6715156

[41] Fung TC, Vuong HE, Luna CDG, Pronovost GN, Aleksandrova AA, Riley NG, Vavilina A, McGinn J, Rendon T, Forrest LR, Hsiao EY. 2019. Intestinal serotonin and fluoxetine exposure modulate bacterial colonization in the gut. Nat Microbiol 4:2064–2073. doi:10.1038/s41564-019-0540-4 31477894 PMC6879823

