# Supplementary material for: Turicibacter sanguinis is a candidate gut microbial pathobiont that promotes metabolic dysfunction-associated steatohepatitis
Source: mSystems. 2026 May 18;11(6):e00292-26. doi: 10.1128/msystems.00292-26 (PMC13289106; doi:10.1128/msystems.00292-26)
Supplement: Supplemental figures — Fig. S1 to S7. [file msystems.00292-26-s0001.docx]

# **Supplementary material**

# **Figure S1**


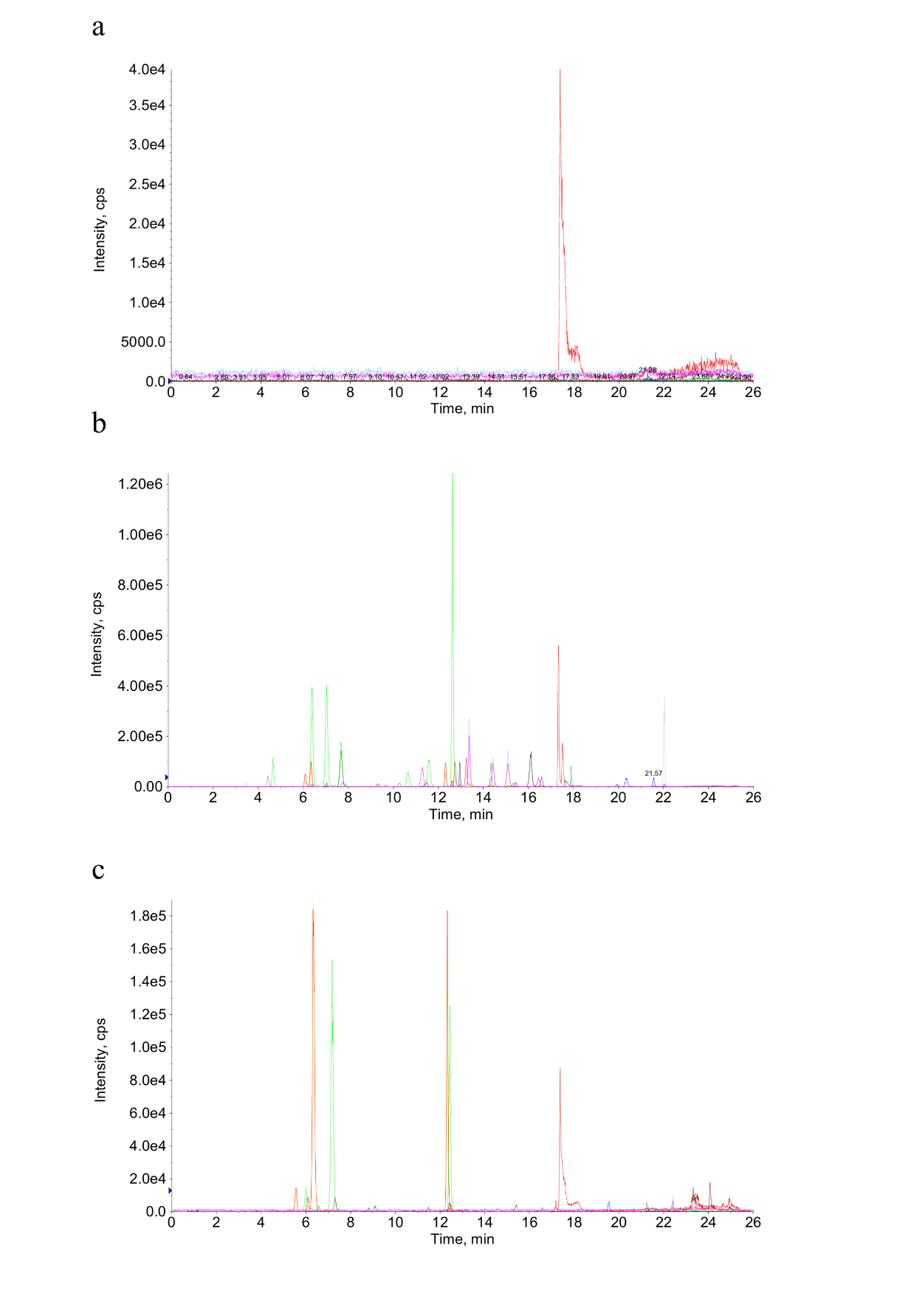


**Fig S1 The representative extracted ion chromatograms of bile acid.** (a) Blank sample; (b) Standard sample; (c) Test sample.

# **Figure S2**

**
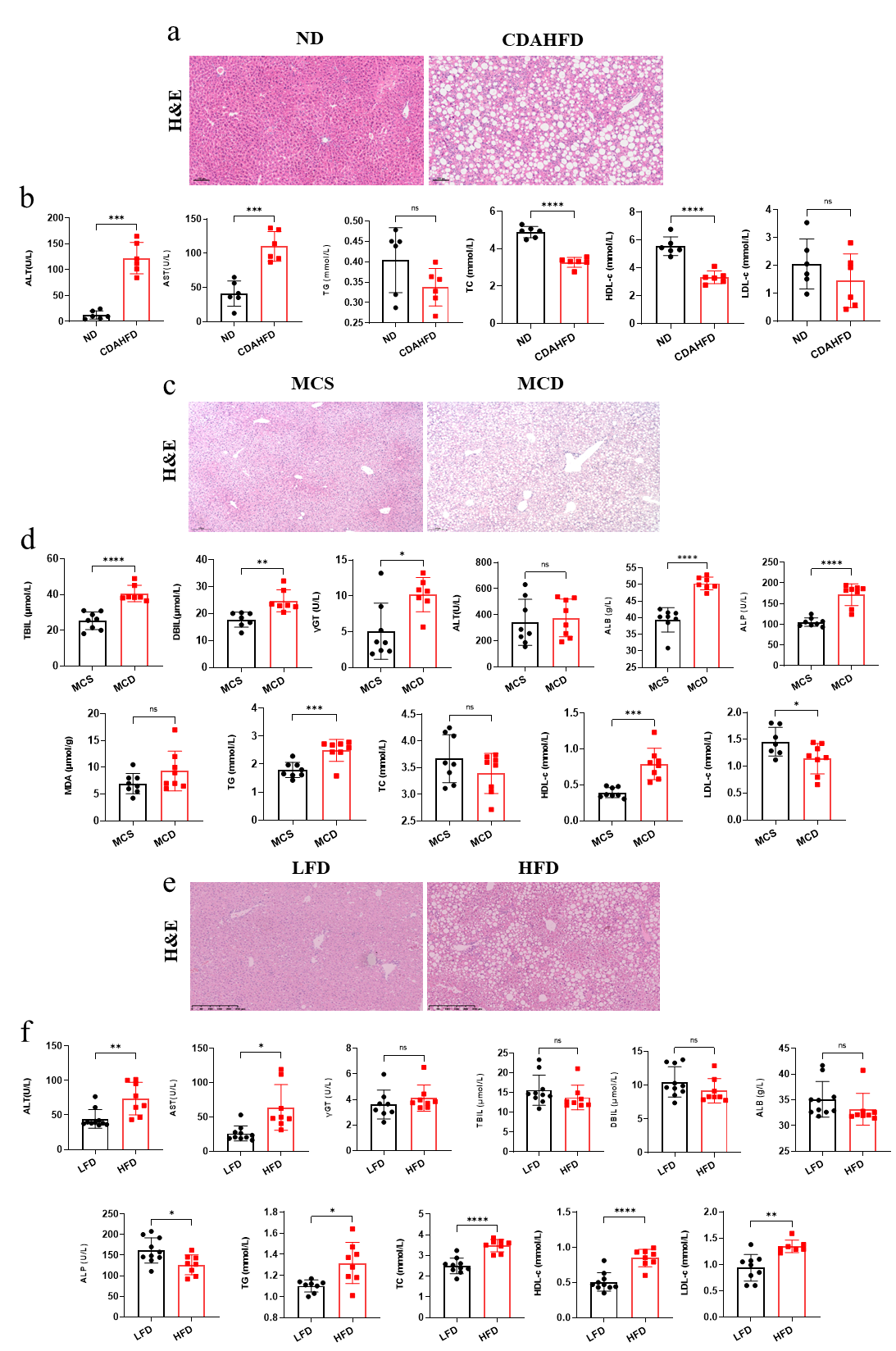
Fig S2 Successful establishment of mouse MASH models.** (a) Representative H&E staining images of liver sections from the CDAHFD model. (b) Serum biochemical parameters of the CDAHFD model, including ALT, AST, TG, TC, HDL-c, and LDL-c; n = 6. (c) Representative H&E staining images of liver sections from the MCD model. (d) Serum biochemical parameters of the MCD model, including TBIL, DBIL, γGT, ALT, ALB, ALP, MDA, TG, TC, HDL-c, and LDL-c; n = 8. (e) Representative H&E staining images of liver sections from the HFD model. (f) Serum biochemical parameters of the HFD model, including ALT, AST, γGT, TBIL, DBIL, ALB, ALP, TG, TC, HDL-c, and LDL-c; n = 8. The CDAHFD model was induced for 8 weeks, the MCD model for 5 weeks, and the HFD model for 18 weeks. Fecal and serum samples were collected at the respective endpoints of model induction. Data are presented as mean ± SD. Statistical differences were analyzed using an unpaired Student’s t-test. *p < 0.05, **p < 0.01, ***p < 0.001, ****p < 0.0001 vs. ND, MCS, LFD, or Healthy group. ns, not significant.

#
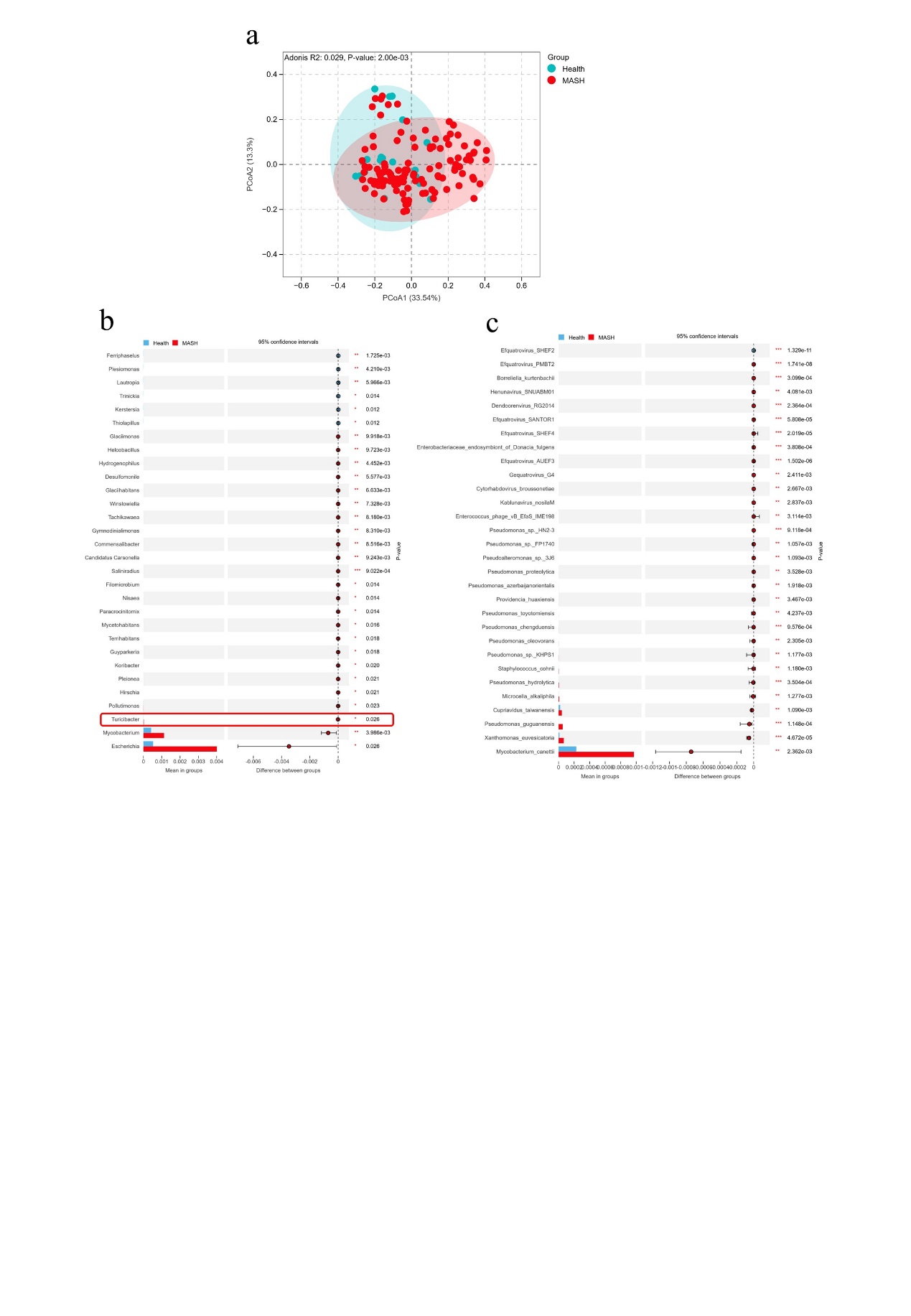
**Figure S3**

**Fig S3** **Changes in the gut microbiota between Healthy individuals and MASH patients.** (a) PCoA plot comparing the gut microbial community structure between Healthy controls and MASH patients. (b) Top 30 differentially abundant bacterial genus. (c) Top 30 differentially abundant bacterial species. Differences were analyzed using the Mann–Whitney U test.

# **Figure S4**


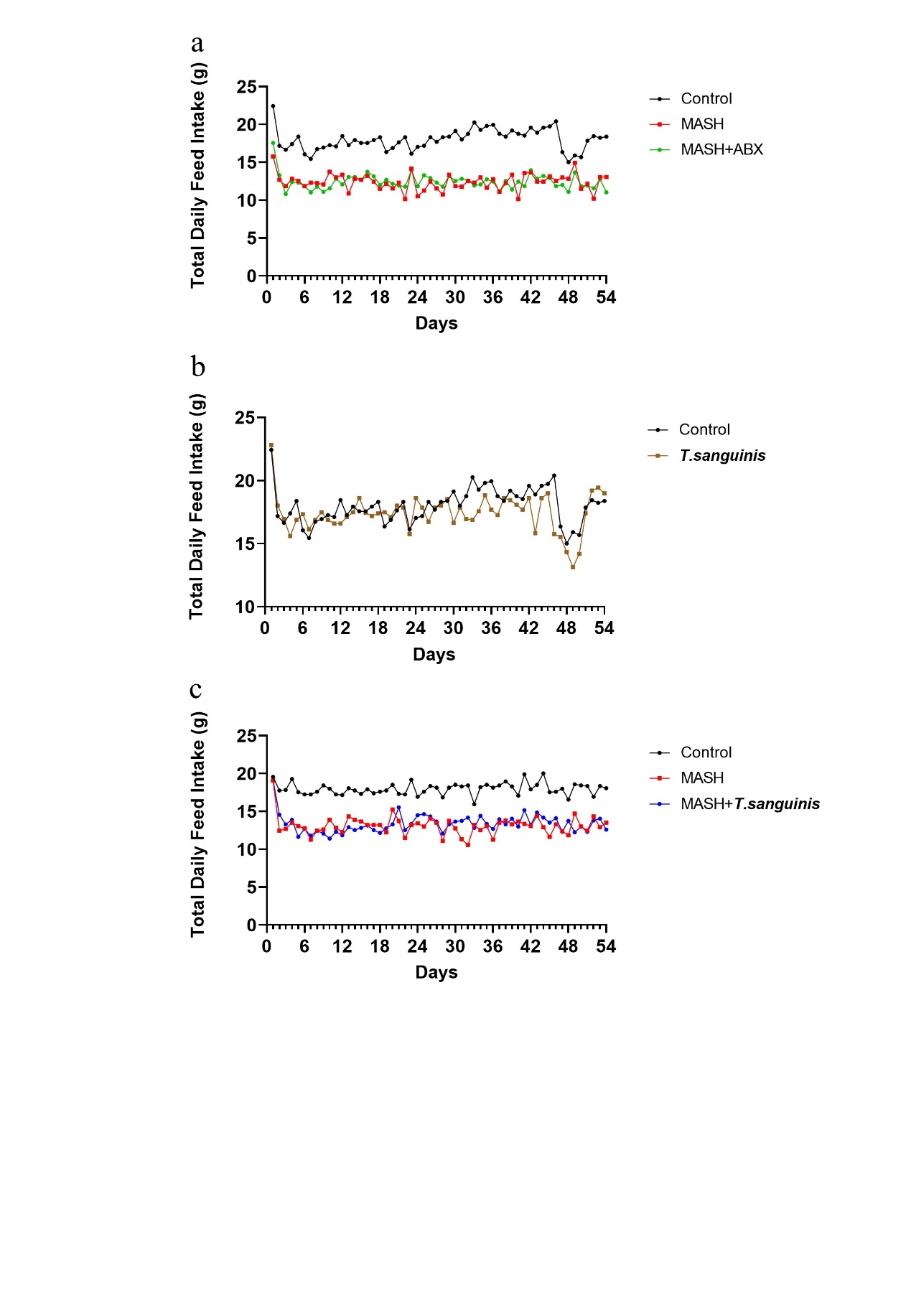


**Fig S4 Daily total food intake of mice in each group.** (a) Daily total food intake in the Control, MASH, and MASH+ABX groups. (b) Daily total food intake in the Control and *T. sanguinis* groups. (c) Daily total food intake in the Control, MASH, and MASH+*T. sanguinis* groups.

# **Figure S5**


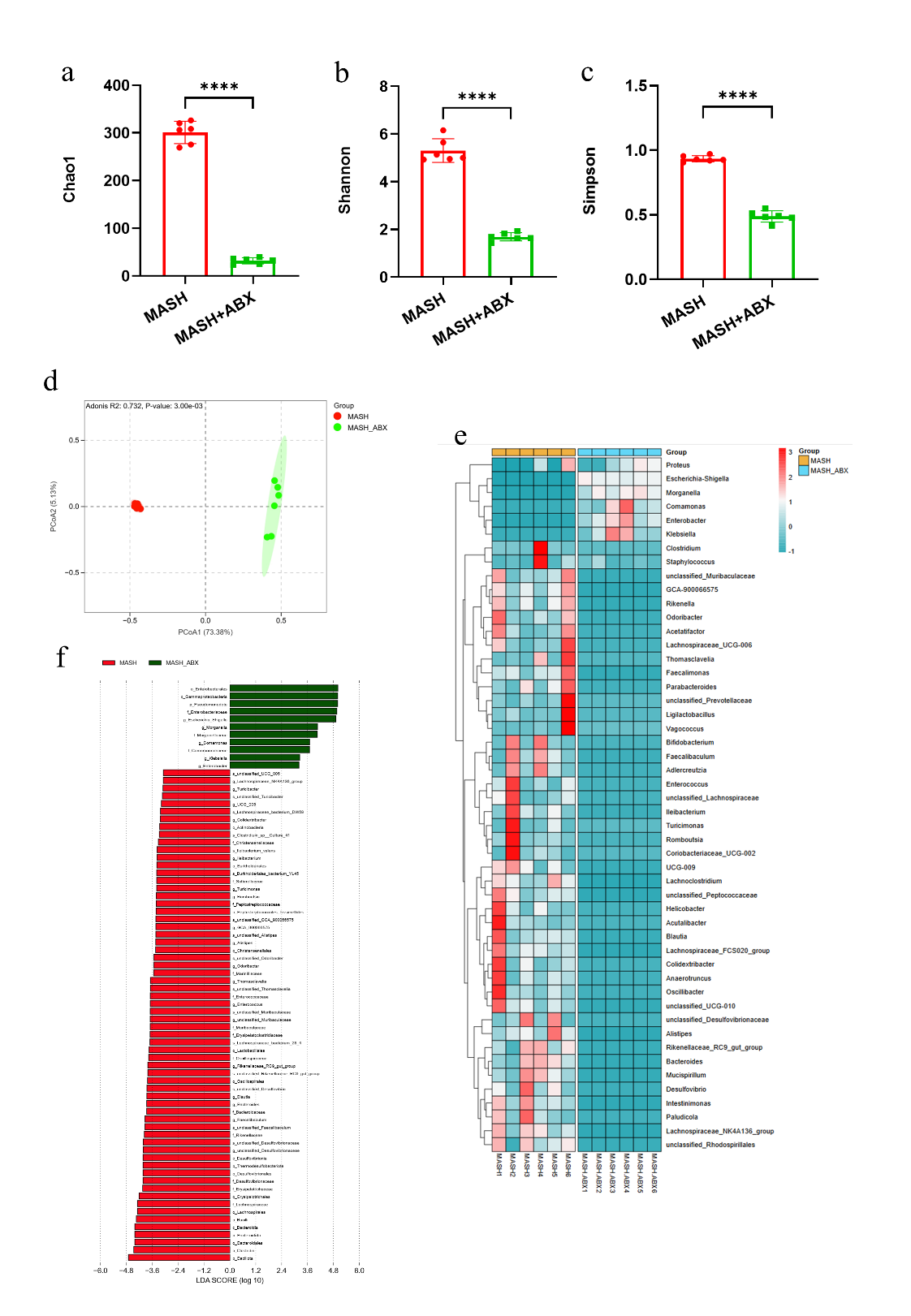


**Fig S5 Changes in gut microbiota composition after antibiotic treatment.** (a–c) Alpha diversity indices of the gut microbiota in each group, including Chao1, Shannon, and Simpson. (d) PCoA of the gut microbial communities in each group. (e) Clustered heatmap showing genus-level relative abundance across all samples (Top 50). (f) LEfSe analysis of the gut microbiota in each group. Data are presented as mean ± SD; n = 6. Differences were analyzed using an unpaired t-test or Welch’s t-test. *****p* < 0.0001 vs the MASH group.

# **
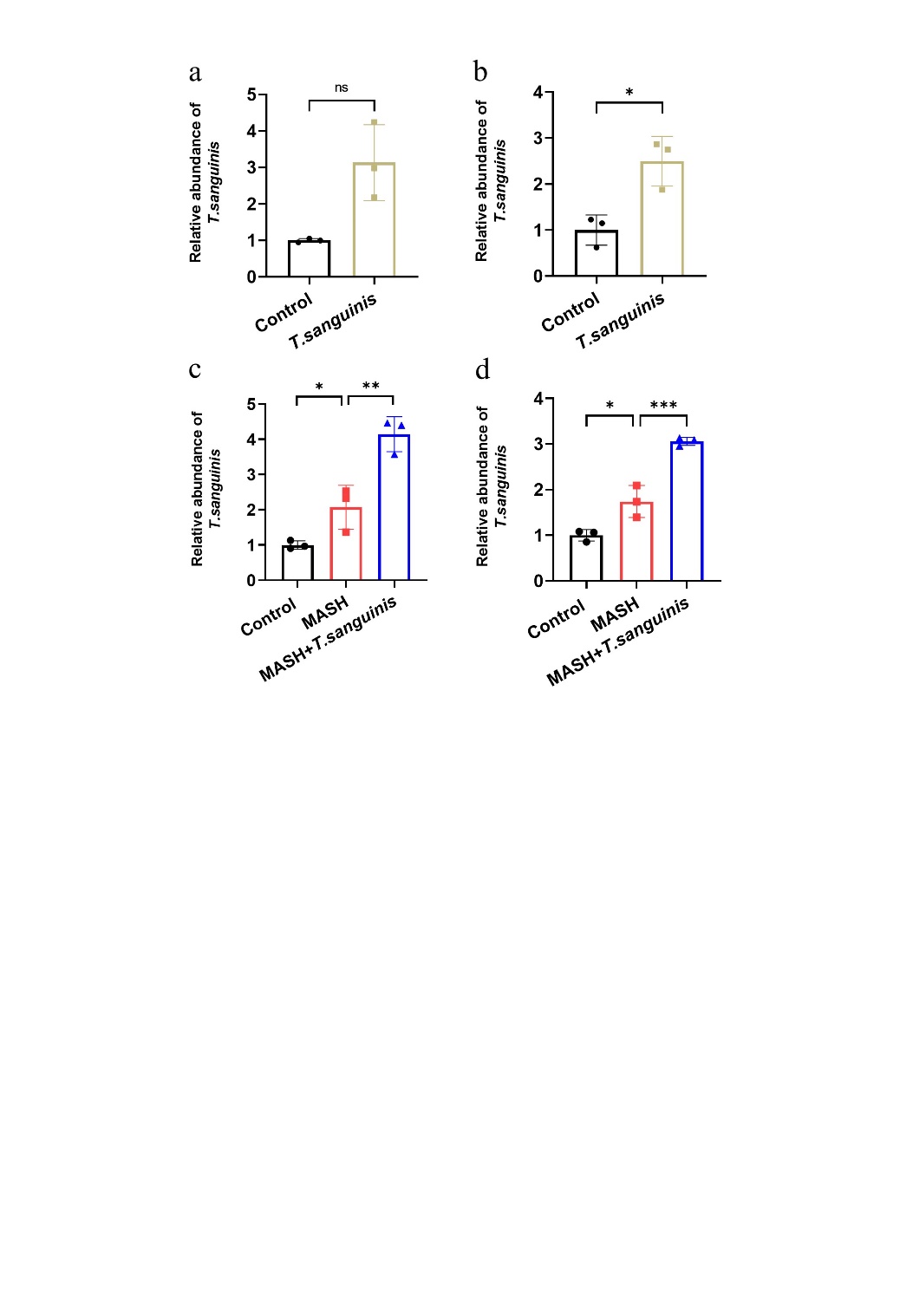
Figure S6**

**Fig S6 The relative abundance of *T sanguinis* was increased in small intestinal and cecal contents.** (a) The relative abundance of *T sanguinis* in small intestinal contents between control and *T sanguinis* group. (b) The relative abundance of *T sanguinis* in cecal contents between control and *T sanguinis* group. (c) The relative abundance of *T sanguinis* in small intestinal contents between between control, MASH and *T sanguinis* group. (d) The relative abundance of *T sanguinis* in cecal contents between control, MASH and *T sanguinis* group. The data are presented as means with SD, n=3. Differences in data were calculated by ordinary one-way ANOVA test. **p* <0.05, ***p* <0.01, ****p* <0.001, vs Control or MASH group.

# **
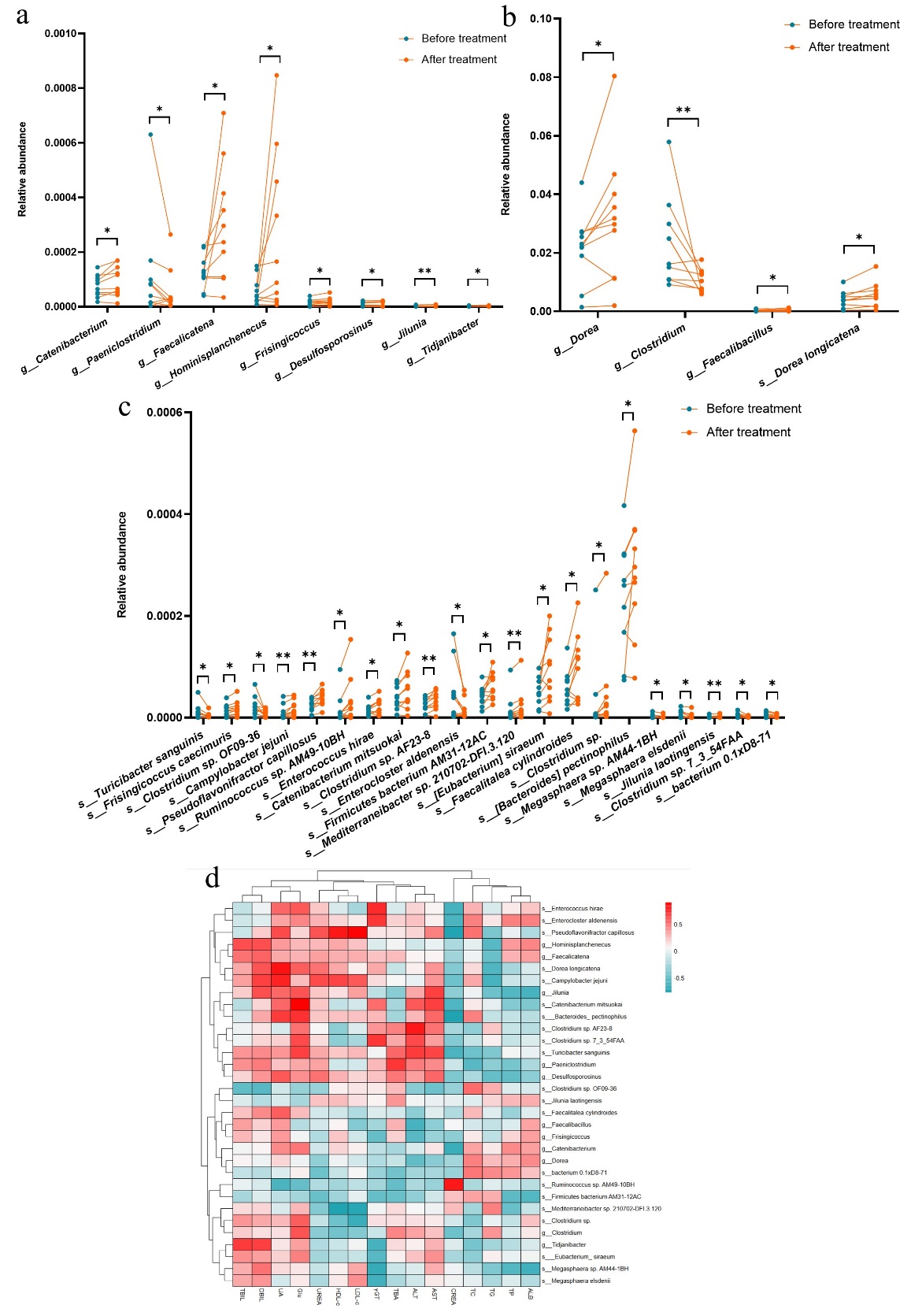
Figure S7**

**Fig S7 Changes in the gut microbiota of MASH patients before and after treatment.** (a–c) Alterations in gut bacterial genus and species in MASH patients before and after treatment. (d) The correlation between the changes in all differential taxa and the changes in clinical biochemical parameters. Data are presented as mean ± SD, n = 10. Differences were analyzed using the Wilcoxon signed-rank test. Correlation analyses were performed using Spearman’s rank correlation. P-values were adjusted for multiple comparisons using the Benjamini–Hochberg FDR method, and the *p*-values displayed in the figures represent FDR-adjusted *p*-values. **p* < 0.05, ***p* < 0.01 vs. the before-treatment group; ns, not significant.
